# Mycobacterial DNA-binding protein 1 is critical for BCG survival in stressful environments and simultaneously regulates gene expression

**DOI:** 10.1038/s41598-023-40941-9

**Published:** 2023-08-29

**Authors:** Amina K. Shaban, Gebremichal Gebretsadik, Mariko Hakamata, Hayato Takihara, Erina Inouchi, Akihito Nishiyama, Yuriko Ozeki, Yoshitaka Tateishi, Yukiko Nishiuchi, Takehiro Yamaguchi, Naoya Ohara, Shujiro Okuda, Sohkichi Matsumoto

**Affiliations:** 1 Department of Bacteriology, School of Medicine, Niigata University, Niigata, Japan; 2 Department of Biology, Assosa University, Assosa, Ethiopia; 3 Department of Respiratory Medicine and Infectious Disease, School of Medicine, Niigata University, Niigata, Japan; 4 Bioinformatics Department, School of Medicine, Niigata University, Niigata, Japan; 5 Toneyama Tuberculosis Research Institute, Osaka Metropolitan University, Osaka, Japan; 6 Center for the Planetary Health and Innovation Science (PHIS), The IDEC Institute, Hiroshima University, Hiroshima, Japan; 7 Department of Pharmacology, Osaka Metropolitan University, Osaka, Japan; 8 Department of Oral Microbiology, Okayama University, Okayama, Japan; 9 Laboratory of Tuberculosis, Institute of Tropical Disease, Universitas Airlangga, Surabaya, East Java Indonesia; 10 Division of Research Aids, Hokkaido University Institute for Vaccine Research & Development, Sapporo, Japan

**Keywords:** Bacteriology, Tuberculosis

## Abstract

Survival of the live attenuated Bacillus Calmette-Guérin (BCG) vaccine amidst harsh host environments is key for BCG effectiveness as it allows continuous immune response induction and protection against tuberculosis. Mycobacterial DNA binding protein 1 (MDP1), a nucleoid associated protein, is essential in BCG. However, there is limited knowledge on the extent of MDP1 gene regulation and how this influences BCG survival. Here, we demonstrate that MDP1 conditional knockdown (cKD) BCG grows slower than vector control in vitro*,* and dies faster upon exposure to antibiotics (bedaquiline) and oxidative stress (H_2_O_2_ and menadione). MDP1-cKD BCG also exhibited low infectivity and survival in THP-1 macrophages and mice indicating possible susceptibility to host mediated stress. Consequently, low in vivo survival resulted in reduced cytokine (IFN-gamma and TNF-alpha) production by splenocytes. Temporal transcriptome profiling showed more upregulated (81–240) than downregulated (5–175) genes in response to MDP1 suppression. Pathway analysis showed suppression of biosynthetic pathways that coincide with low in vitro growth. Notable was the deferential expression of genes involved in stress response (*sigI*), maintenance of DNA integrity (*mutT1*), REDOX balance (*WhiB3*), and host interactions (*PE/PE_PGRS*). Thus, this study shows MDP1’s importance in BCG survival and highlights MDP1-dependent gene regulation suggesting its role in growth and stress adaptation.

## Introduction

Over the years *Mycobacterium tuberculosis* (Mtb) has developed into a formidable pathogen. It was the leading cause of human death from a single infectious agent before SARS-CoV-2 emergence. In 2021, there were an estimated 10.6 million new tuberculosis (TB) cases and 1.6 million deaths mainly due to antimicrobial resistance, making TB a global health concern^1, 2^. *Mycobacterium tuberculosis* variant Bacillus Calmette-Guérin (BCG), the sole licensed vaccine against TB, is reported to provide protection against childhood TB in vaccinated children^3, 4^. On the other hand, studies have also reported varying BCG protective efficacy (ranging from nil to 80%) against pulmonary TB in vaccinated adults, thus heightening current efforts to develop a more effective vaccine^4–6^. However, no component vaccine in clinical trials has been reported to surpass BCG, which makes the development of novel BCG-based TB vaccines a viable option. Aside from TB, BCG has immunostimulatory effects that offer protection against unrelated pathogens including viral infections^7, 8^ and has 80% effectiveness in preventing recurrence of bladder cancer^9^. Most recently, recombinant BCG strains expressing antigens from pathogenic bacteria and viruses including SARS-CoV-2 are being produced for vaccine development purposes^10, 11^.

Since BCG is a live attenuated vaccine, its viability is considered a precondition for the induction of effective immunity against Mtb^4, 12–14^. It can infect organisms and confer long-lasting immune response for up to 10–15 years^5, 15^, and is a safe model for virulent Mtb^5, 16, 17^. A common feature between Mtb and BCG is their ability to adapt to harsh environments within the host resulting in growth and long-term survival. This adaptation ability complicates Mtb elimination^18^ but promotes BCG’s protective effectiveness against TB, and other unrelated pathogens due to its non-specific protective effects^12–14^. Understanding this underlying adaptation mechanism will thus help inform the development of more effective anti-TB vaccines^4^.

Adaptation to harsh and changing environments by organisms is attributed to precise gene regulation controlled by a multitude of proteins^19^. These include nucleoid associated proteins (NAPs): a group of small but abundant DNA binding proteins that organize DNA into compact structures and influence transcription^20–23^. Through DNA association, NAPs facilitate rapid and appropriate responses to a variety of stress factors–including host defense mechanism, radiation, oxidative stress or nutrient exhaustion–which is crucial for cell survival^24^. In mycobacteria, the Mycobacterial DNA binding protein 1 (MDP1) coded by *hupB* (also *hlp)* gene, is a histone-like NAP conserved among all tested mycobacterial species. It is considered essential in pathogenic slow growing mycobacteria like BCG and Mtb^20^, and dispensable in non-pathogenic rapid grower *M. smegmatis*^25, 26^. Gene disruption and mutation analysis have shown highly diverse phenotypes that highlight MDP1’s importance in mycobacterial species physiology and adaptation in diverse microenvironments. Key among its roles is in enabling different Mycobacterium species to survive in macrophages where it aids in iron scavenging^27^, tolerance to low pH^28^, drug resistance^29, 30^, and immune modulation^31^.

Structurally, MDP1 is a dimeric protein with an N-terminal domain that directly interacts with DNA, and a C-terminal domain that facilitates DNA sequence-specific binding^20, 23, 32^. The C-terminal has an intrinsically disordered region (IDR) that participates in DNA protection and compaction^26^. Together, these domains facilitate MDP1 binding to AT-rich sequences which are mainly localized at promoter regions^33^, implying a global reach and influence on gene expression^20, 23, 32^, that we theorize facilitates its multifaceted roles. Additionally, MDP1 interacts with other NAPs to carry out its functions. Most recently, the Lsr2 NAP, was shown to interact with MDP1 forming a complex that influences DNA architecture and regulates transcription^23^. However, due to the limitations of qPCR technologies, knowledge on the extent of MDP1 gene regulation has so far been limited to a few genes including siderophore genes in iron deficient environments^27^ and *katG* conferring isoniazid resistance^29^. As such, utilizing an essential gene conditional-knockdown system, by combining CRISPR-dCas9 and tetracycline-controlled transcriptional activation system, we evaluate MDP1’s role in BCG growth and survival under stress in vitro and in vivo. We further employ next generation sequencing to decipher the genome-wide transcription regulation by MDP1 in BCG, which might uncover MDP1-dependent adaptation mechanism.

## Results

### MDP1 suppression lowers BCG growth in vitro

**Figure 1:**
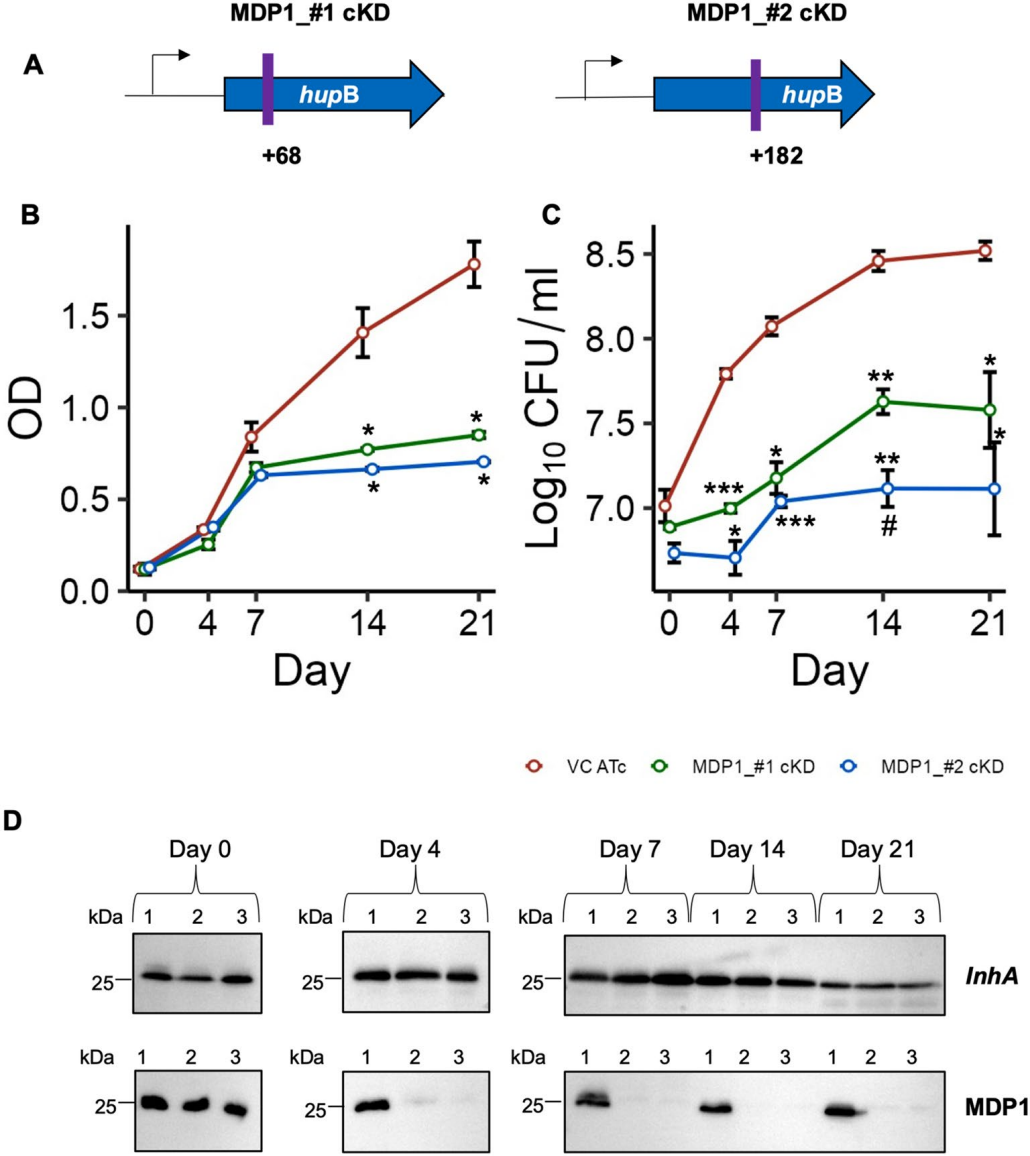
Growth kinetics of BCG MDP1 cKD (**A**) Position of *hupB* target sequence start site shown by purple vertical lines for MDP1_ #1 and MDP1 #2 cKD BCG. Numbering indicates first nucleotide on the target sequence relative to annotated start coding sequence. Arrow indicates position of transcription start site. BCG growth kinetics depicted by (**B**) optical density (OD_600_ nm) and (**C**) CFU over 21 days. Bacteria were cultured in 7H9/ADC medium supplemented with 200ng/ml ATc every 48 hours to induce sgRNA expression. At indicated time points culture aliquots were harvested to determine the OD and enumerate CFU after culturing on 7H10/OADC agar. Data represent mean ± SE from three biological replicates. Statistical differences between VC ATc and MDP1-cKD BCG were assessed by unpaired Welch’s *T* test, *P<0.05 **P<0.01 ***P<0.001; ^#^p<0.05 indicate differences between MDP1_#1-cKD and MDP1_#2-cKD. (**D**) Representative western blot images cropped from different gels delineated with black border lines confirming the expression of MDP1: *InhA* serves as a loading control. Full-length blot images in Supplementary Fig. 3. 1-VC ATc, 2-MDP1_#1-cKD, 3-MDP1_#2-cKD. Image is representative of three experiments.

To assess the roles of MDP1 in BCG, we constructed MDP1 conditional knockdown BCG Tokyo 172 by using CRISPR/dCas9 system targeting two sites on the MDP1 gene (MDP1_#1 and MDP1_#2) (Fig. 1A), to compare phenotype similarities and hence confirm same gene target^34, 35^. We confirmed that MDP1 suppression caused low BCG growth over a period of 21 days*.* By day 14 there was a ~2-fold difference in cell density (OD 600nm) of the MDP1 conditional knockdown (cKD) BCG (MDP1-cKD BCG), compared to vector control (VC) (Fig. 1B). MDP1 suppression resulted in significantly lower growth of ~0.8 and 1 log_10_ colony forming units (CFUs) for MDP1_#1 and MDP1_#2 cKD respectively, compared to VC from day 4. Surprisingly, we also observed a significant difference of ~0.5 log_10_CFU between MDP_#1-cKD and MDP1_#2-cKD at day 14 (Fig. 1C). These growth differences in comparison to VC corresponded to MDP1 suppression from day 4 to 21, as evidenced by undetectable MDP1 bands in MDP1-cKD BCG on western blot (Fig. 1D). *InhA* was used as a loading control. We confirmed that these differences resulted from induction of sgRNA and dCas9 expression by ATc and not by the diluent DMSO as VC and DMSO treated MDP1-cKD BCG showed no difference in neither their MDP1 levels nor growth (Supplementary Fig. 1). This data supports reports on MDP1 essentiality in BCG^29, 36, 37^, as suppression observed during the initial 4-day period immediately resulted in significantly low growth. Contrary to our findings, a previous study reported that MDP1 suppression led to accelerated BCG growth in vitro as quantified by ATP levels^28^. However, we did not observe any significant difference in ATP levels between VC and MDP1-cKD BCG (Supplementary Fig. 4).

### MDP1 is involved in intrinsic tolerance to 1st line TB drugs

MDP1 deficient *M. smegmatis* was previously shown to have elevated levels of ATP^25^, unlike MDP1-cKD BCG (Supplementary Fig. 4). This might imply less influence on ATP metabolism by MDP1 in BCG, and that MDP1-cKD BCG might be less affected by energy perturbations. As such, we tested the effect of bedaquiline, which blocks ATP synthesis by binding to ATP synthase^1, 38^, on the mutant survival.

**Figure 2:**
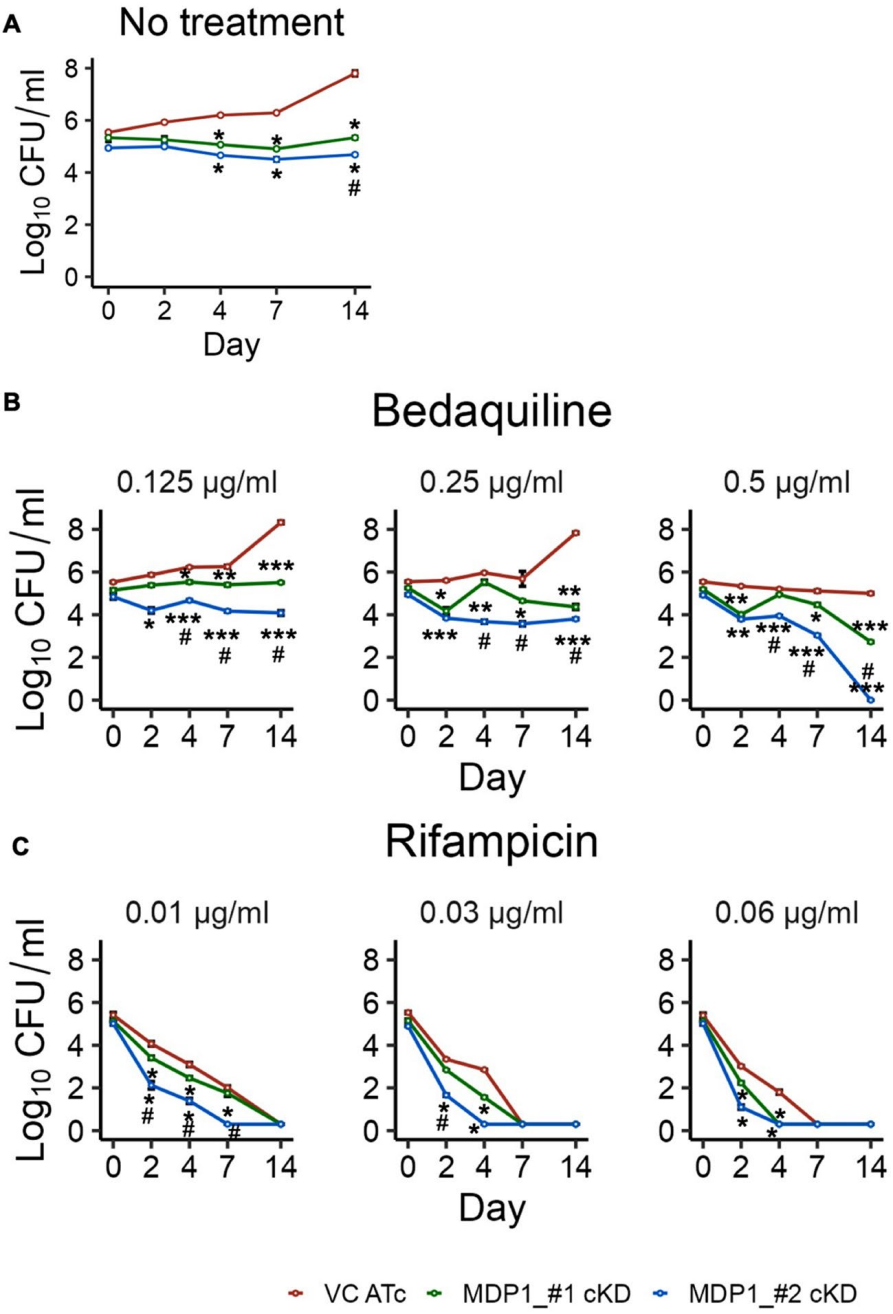
Susceptibility of MDP1-cKD BCG to antibiotics. CFU quantification of indicated strains with (**A**) no treatment, after exposure to (**B**) bedaquiline and (**C**) rifampicin as a positive control, over 14 days. After confirmation of MDP1 expression in VC and suppression in MDP1-cKD BCG, exponential phase cultures were harvested, and OD adjusted to 0.002 followed by exposure to different drug concentrations in 7H9/ADC medium. MDP1 expression was maintained by supplementing cultures with 200ng/ml ATc every 48 hours. Data represent mean ± SE from three biological replicates. Statistical differences between VC ATc and MDP1-cKD BCG were assessed by unpaired Welch’s *T* test, *P < 0.05 **P < 0.01 ***P < 0.001; ^#^p<0.05 indicate differences between MDP1_#1-cKD and MDP1_#2-cKD.

As expected, there was significant difference (p < 0.05) between the no drug treated VC and MDP1-cKD from day 4 to14 (Fig. 2A). Bedaquiline treatment showed a dose dependent killing with exposure to 0.125 and 0.25 µg/ml showing no change in CFU for MDP1-cKD BCG, while the VC increased by 2-fold between day 7 and 14 of drug exposure (Fig. 2B). However, at 0.5 µg/ml VC maintained its growth level while MDP1-cKD BCG showed gradual decrease in CFU up to day 7. At day 14, MDP1 #1 cKD had 3-fold reduction in CFU while MDP1 #2 cKD mutant was sterilized. From day 2 to 14 MDP-cKD CFU was significantly different from VC at all drug concentrations. Surprisingly, we also observed significant differences between MDP1 #1 cKD and MDP1 #2 cKD with increase in bedaquiline exposure time and concentration.

Rifampicin, which inhibits RNA polymerase and hence gene transcription^39^, was used as a positive control. Exposure to 0.01µg/ml rifampicin resulted in sterilization of MDP1_#2-cKD by day 7, while MDP1_#1-cKD and VC were sterilized by day 14 (Fig. 2C). Increase in Rifampicin concentration resulted in sterilization of all bacteria by day 7 of drug exposure. The increased bacterial death in drug treated cultures supplemented with ATc and not in the untreated but ATc supplemented cultures proves that the death is caused by BCG sensitivity to the drugs and not ATc. Differences between MDP1_#1 and MP1_#2-cKD upon drug exposure suggests possible clonal variation with regards to the mutant’s response to drugs. Similar unexplained clonal variation was observed in a previous study where Mtb mutants having > 80% suppression of an essential gene exhibited different antibiotic sensitivity^40^. Overall, our results indicate that MDP1 impacts on the ability of BCG to respond to energy constrains, with stronger inhibition of ATP production being bacteriostatic to VC, but bactericidal to MDP1-cKD BCG.

### MDP1 suppression reduces tolerance to oxidative stress

**Figure 3:**
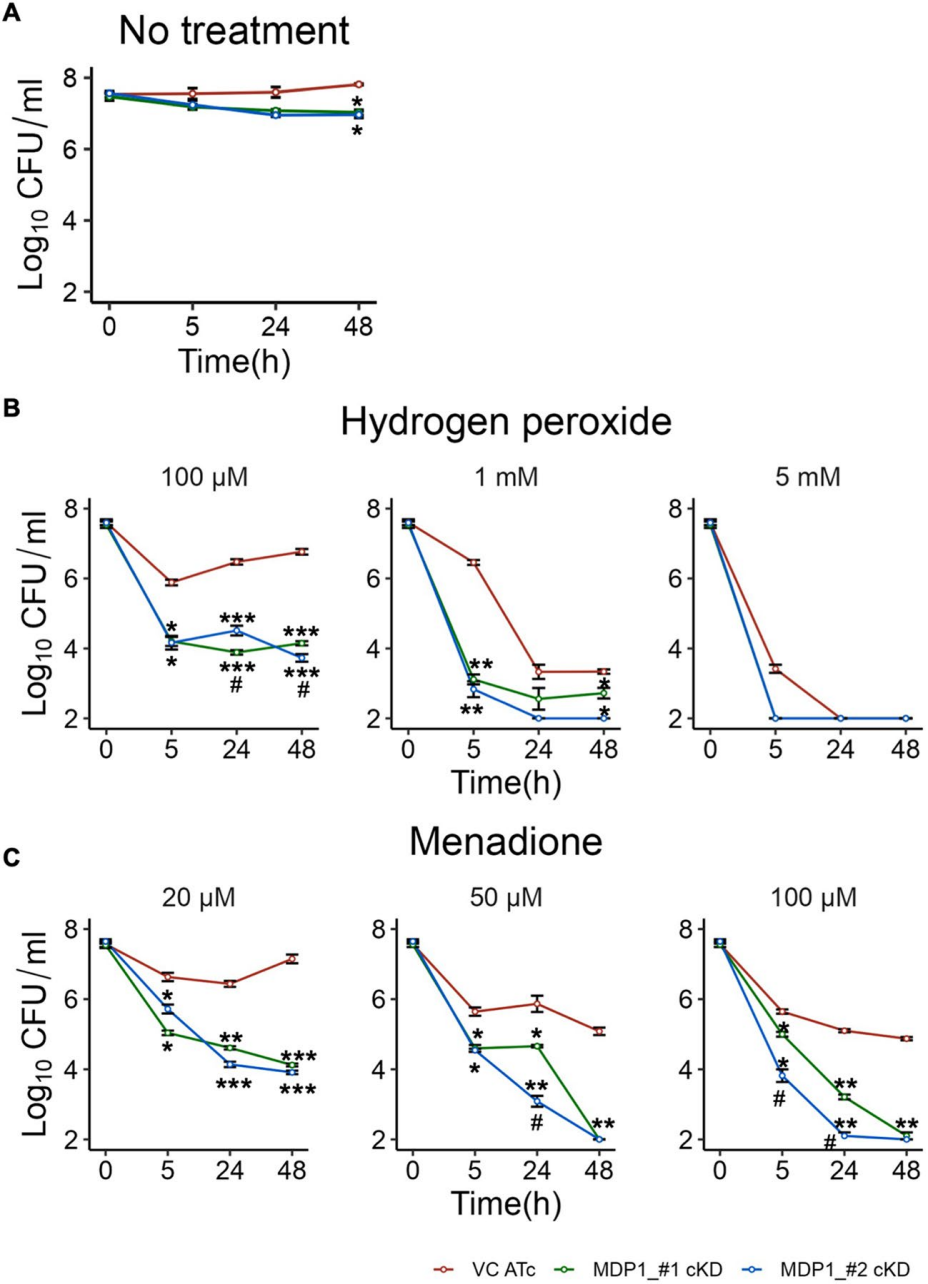
Oxidative stress tolerance of MDP1-cKD BCG. CFU quantification of indicated strains with (**A**) no treatment, and after exposure to (**B**) hydrogen peroxide (**C**) menadione over 48 hours. After confirmation of MDP1 expression in VC and suppression in MDP1-cKD BCG, exponential phase cultures were harvested, and OD adjusted to 0.02 followed by exposure to different concentrations of oxidizing agents in 7H9/ADC medium. MDP1 suppression was maintained by supplementing cultures with 200ng/ml ATc at day 0. Data represent mean ± SE from three biological replicates. Statistical differences between VC ATc and MDP1-cKD BCG were assessed by unpaired Welch’s *T* test, *P < 0.05 **P < 0.01 ***P < 0.001; ^#^p < 0.05 indicate differences between MDP1_#1-cKD and MDP1_#2-cKD.

MDP1 has been shown to protect mycobacterial DNA from oxidative damage via Fenton reaction^41^. In Mtb, expression of MDP1 increased when the bacilli were exposed to oxidative stress which shows its importance under such conditions^39^. To test the role of MDP1 in BCG oxidative stress adaptation, we compared the survival of the mutants in sub-lethal and lethal concentrations of hydrogen peroxide and menadione^25, 42^. As expected, there was significant difference between the untreated VC and MDP1-cKD at 48 hours (Fig. 3A). MDP1-cKD BCG were highly susceptible to the lowest concentrations (100 µM) of hydrogen peroxide with up to 4-log reduction in CFU after 5 hours exposure, as opposed to the 2-log reduction in VC (Fig. 3B). However, VC was able to increase its growth after 5 hours exposure compared to the stagnant growth observed in MDP1-cKD BCG. All cultures were susceptible to lethal concentrations (1mM and 5 mM) of hydrogen peroxide.

Exposure to 20 µM menadione resulted in 4-log reduction in the CFU of MDP1-cKD BCG by 48 hours (Fig. 3C). On the other hand, at 20 µM menadione VC was able to adapt after 2-log reduction at 24 hours and resumed growing by 48 hours to almost the level of the untreated VC (Fig. 3A). At 50 µM menadione, there was a sharp decline in the viability of MDP1-cKD BCG which were sterilized by 48 hours. In contrast, VC managed to survive with only 2-log reduction in CFU. Higher concentrations of menadione (100 µM) resulted in sterilization of MDP1_#2-cKD by day 24, while MDP1_#1-cKD was sterilized at 48 hours. VC on the other hand still managed to maintain its viability up to 48 hours after a 2-fold reduction in CFU at 5 hours. Therefore, compared to VC and the no treatment controls, MDP1-cKD BCG, although with some variation, were highly susceptible to oxidative stress. Thus, our results highlight the importance of MDP1 in BCG survival under oxidative stress, which concurs with a study that evidenced significantly reduced MTb ∆MDP1 growth in hydrogen peroxide compared to MDP1 expressing controls^39^.

### Low infectivity and survival of MDP1-cKD BCG in macrophages and mice

**Figure 4:**
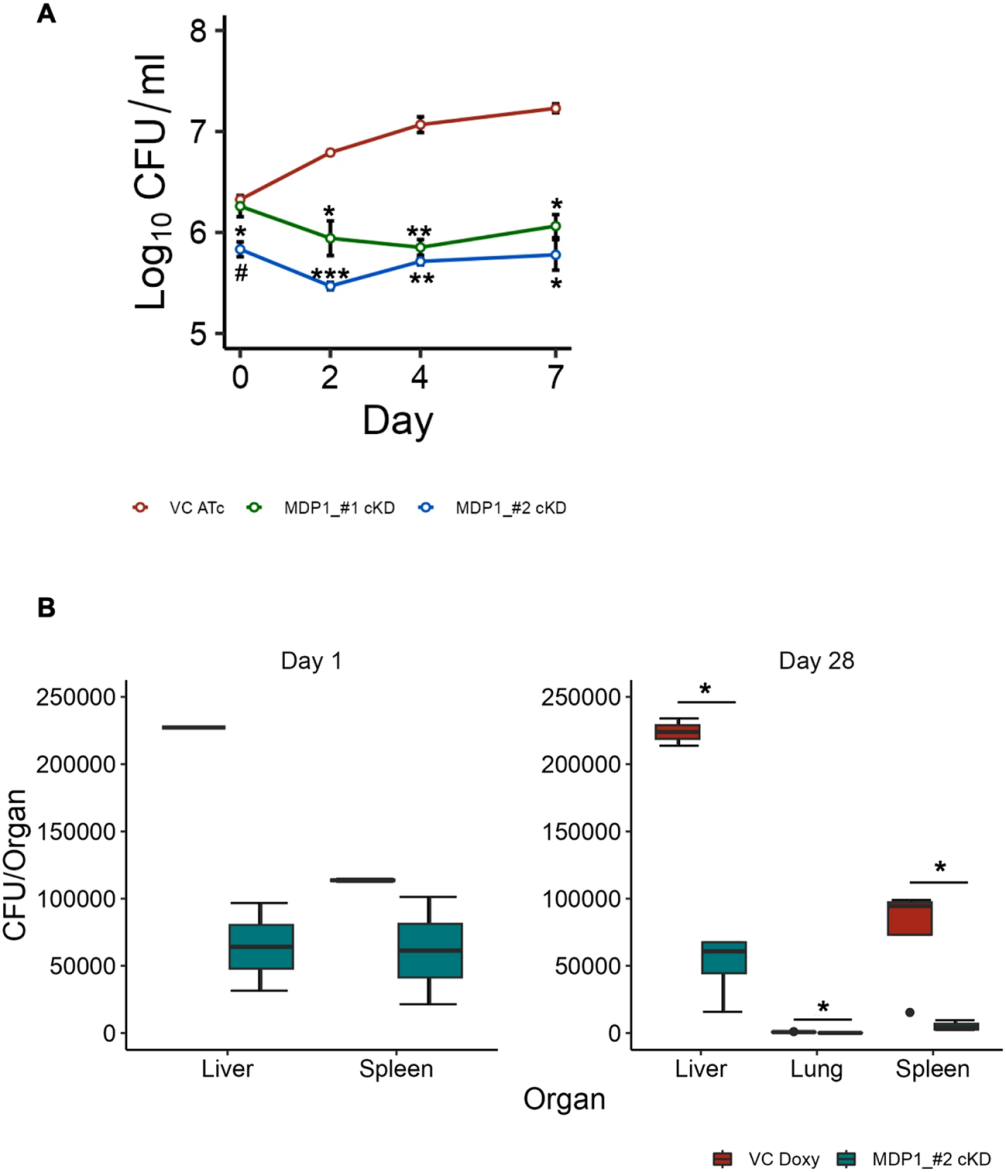
Survival of BCG MDP1 cKD in macrophages and in vivo (**A**) Intracellular survival of MDP1-cKD BCG compared to VC, over 7 days. After confirmation of MDP1 expression in VC and suppression in MDP1-cKD BCG, THP-1 cells were infected with indicated strains at a multiplicity of infection (MOI) of 1:1 for 4 hrs. The cultures were subsequently washed twice with warm serum-free DMEM to remove uninfected BCG and finally cultured in DMEM supplemented with 200ng/ml ATc every 48 hrs. The cells were incubated at 37 °C in a humidified 5% CO_2_ incubator. BCG CFU was determined at indicated timepoints. Data represent mean ± SE from three biological replicates. Statistical differences between VC ATc and MDP1-cKD BCG were assessed by unpaired Welch’s *T* test, *P < 0.05 **P < 0.01 ***P < 0.001; ^#^p < 0.05 indicate differences between MDP1_#1-cKD and MDP1_#2-cKD. (**B**) *In vivo* survival of indicated strains over 28 days. Female C57BL/6J mice (n = 2–8) were intraperitoneally infected with 5 × 10^6^ CFU of indicated BCG strains, suspended in 200 µl PBS. MDP1 expression was maintained by supplementing mice drinking water with 20 µg/ml Doxycycline (Doxy). BCG CFU was quantified from harvested mice organ homogenates plated on 7H10/OADC agar at indicated time points. Data represent mean ± SE from 2 to 8 mice per group. Statistical differences were analyzed using Mann-Whitney *U* test, *P < 0.05

Fitness of a gene varies in different environments; therefore, gene essentiality in vitro does not necessarily translate to essentiality in a host cell or in vivo^43, 44^. As such, to test how MDP1 suppression influenced BCG survival in host environments, we first assessed the ability of MDP1-cKD BCG to survive in macrophages, which is the first line of host defense encountered by BCG before establishing infection^28^. We infected human leukemic macrophage cell line THP-1 with VC and MDP1-cKD BCG at an MOI of 1 and quantified growth by CFU enumeration. The infectivity of MDP1_#2-cKD was lower than VC and MDP1_#1-cKD (Fig. 4A). While VC growth increased over the 7-day culture period, MDP1-cKD BCG had significantly reduced CFU at day 2 which remained constant by day 4 followed by a slight increase at day 7. By the 7th day, VC growth had increased 1 log as opposed to the less than 0.5 log increase by both MDP1-cKD BCG. Taken together, this shows that MDP1 suppression might not only lower the infectivity of BCG, but also its growth in macrophages.

Next, we evaluated the role of MDP1 in BCG in vivo survival. We intraperitoneally infected mice with VC and MDP1_#2-cKD and quantified CFU as a measure of survival. At day 1, VC infected mice had 4 times more CFU counts than MDP1_#2-cKD infected mice as seen from the ~120,000 liver CFU count difference. A similar trend was observed in the spleen with VC infected mice having 2 times the CFU count of MDP1_#2-cKD infected mice. The VC infected mice maintained the bacterial load in the liver and spleen by day 28. This was contrary to the decrease in bacterial load observed in MDP1_#2 infected mice. The differences in liver, lung and spleen CFUs between VC and MDP1_#2-cKD infected mice at day 28 were significant (Fig. 4B). Of note is that there was prolonged survival of VC as opposed to MDP1_#2-cKD. This suggests that MDP1 may be important in establishing infection and facilitating BCG in vivo survival.

### Deficient immune response of splenocytes from MDP1-cKD immunized mice

Prolonged in vivo survival of BCG is important for continuous immune stimulation^17^. We next determined how the viability of MDP1_#2-cKD will impact mice immune response. We compared Th1 immune response elicited in VC, MDP1_#2-cKD and no immunization (NI) control mice groups 4-weeks post-immunization.

**Figure 5:**
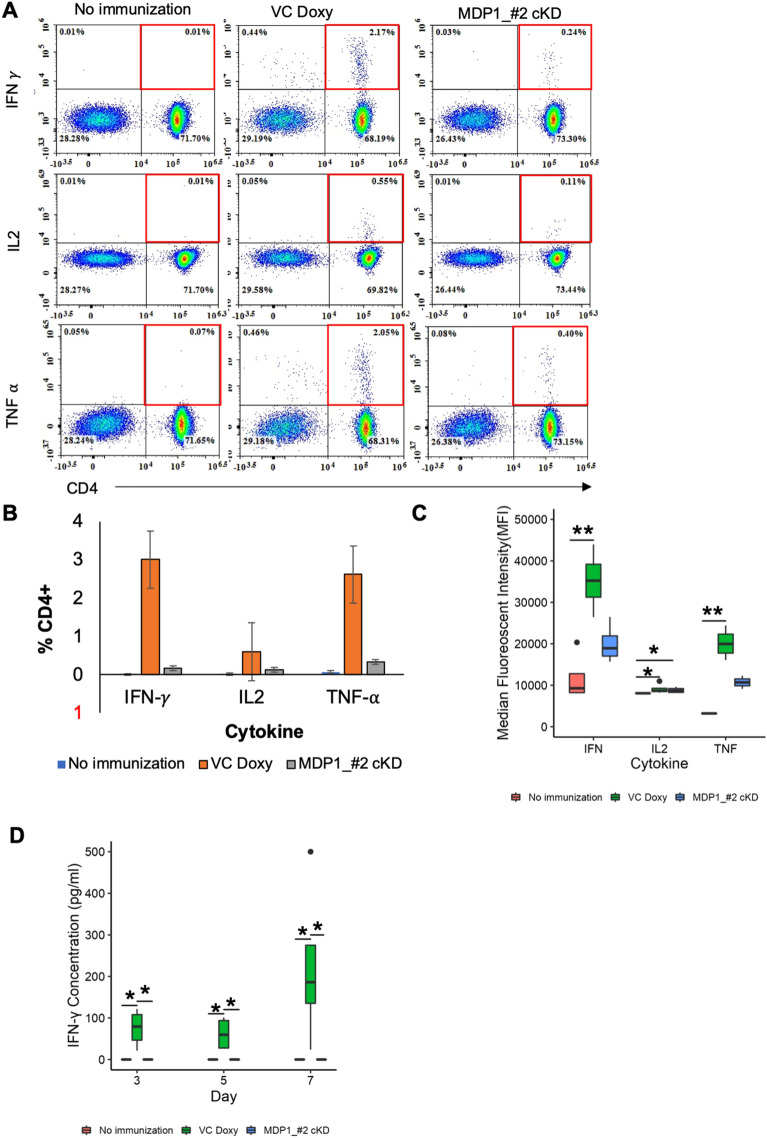
Influence of BCG viability on mice splenocyte immune response (**A**) Representative density plots showing intracellular IFN-γ, IL2 and TNF-α staining following 12 hr PPD stimulation of mice splenocytes. Four weeks prior, mice were intraperitoneally immunized with VC, MDP1_#2-cKD BCG or saline for the no immunization control group. Red boxes indicate populations of cytokine producing CD4^+^ T cells from each immunized group. (**B**) Summary bar graph depicting the percentage of CD4^+^ cytokine^+^ population presented as mean ± SD from 4-6 mice per group. (**C**) Intracellular cytokine MFIs of T cells in each immunized mice group. (**D**) IFN-γ quantification by ELISA following PPD stimulation of immunized mice splenocytes over 7 days. Data (**B**) presented as mean ± SE from 4 to 6 mice. Data (**C** and **D**) presented as box and whisker plots with max to min displayed. Statistical significance was determined using Kruskal-wallis with Dunn multiple comparison test for (**C**) and (**D**). *P < 0.05 **P < 0.01.

Generally, purified protein derivative (PPD) stimulated mice splenocytes from the 3 mice groups showed higher CD4^+^cytokine^+^ than CD8^+^cytokine^+^ population (Supplementary Fig. 5). From the CD4^+^ cells, the VC immunized mice had higher cytokine^+^ population (IFN-γ-3.01%, IL2-0.60% and TNF-α-2.62%) than the MDP1_#2-cKD (IFN-γ-0.17%, IL2-0.60% and TNF-α-0.33%) and the NI (all less than 1%) immunized mice (Fig. 5A,B). Intracellular cytokine quantification using mean fluorescence intensity (MFI) showed that the immunized mice groups had significantly higher IFN-γ production followed by TNF-α then IL2 (Fig. 5C). IFN-γ and TNF-α production were significantly higher in VC than NI group, while IL2 production was significantly lower in the NI control than VC and MDP1_#2-cKD immunized mice. To validate the flow cytometry results we quantified IFN-γ production by ELISA which confirmed significantly higher IFN-γ production by splenocytes from the VC immunized group compared to MDP1_#2-cKD and the NI group (Fig. 5D). The quantity of IFN-γ also increased with increase in PPD incubation time for VC but remained the same for both NI and MDP1_#2-cKD immunized mice. This suggests that MDP-dependent survival of BCG influences Th1 immune response in mice.

### RNA seq identify MDP1 as a gene repressor

**Figure 6:**
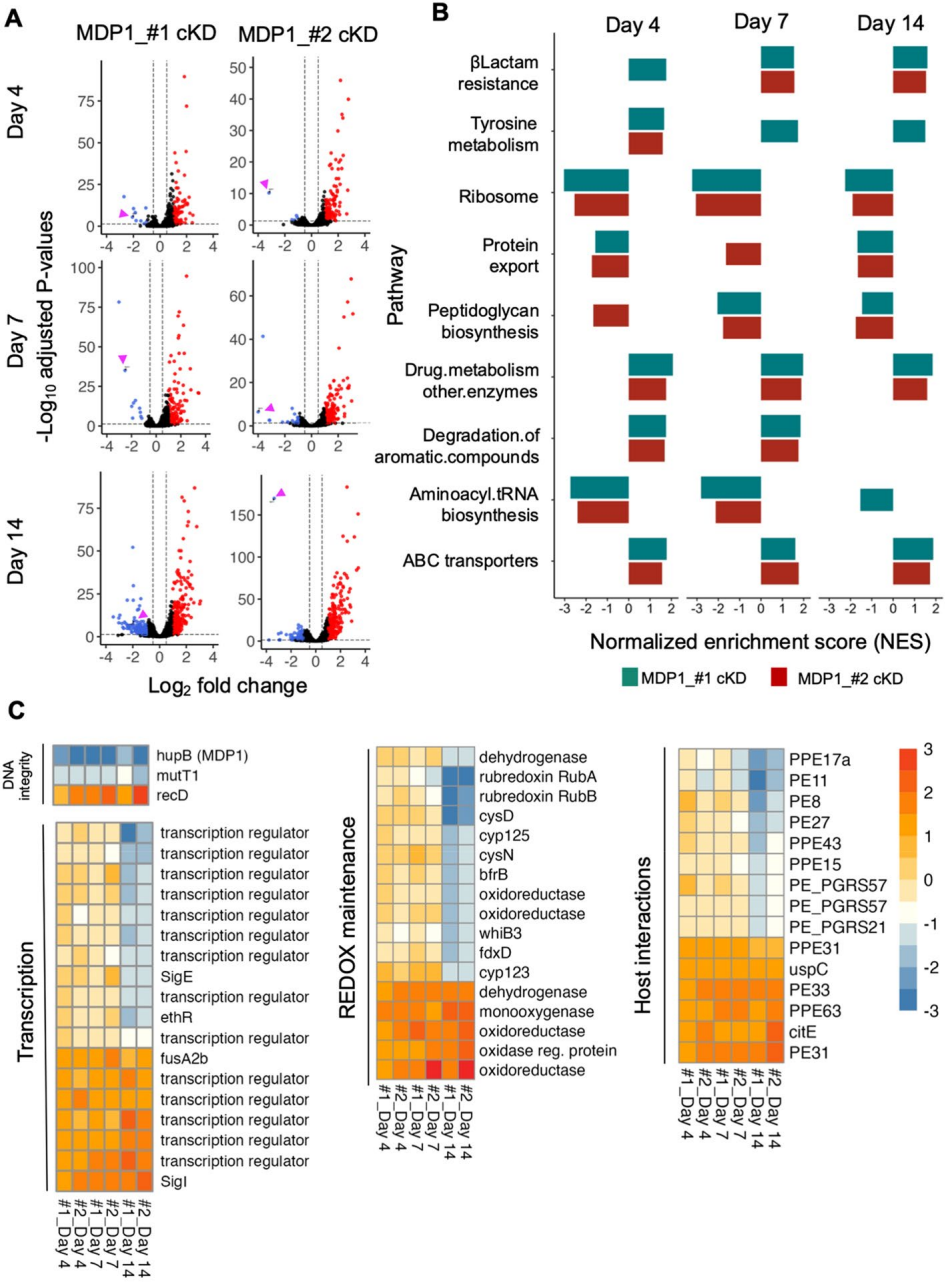
Global transcription response to MDP1 suppression in BCG (**A**) Global gene expression changes in MDP1-cKD BCG compared to VC ATc at day 4, 7 and 14, from triplicate experiments. Genes with adjusted p-value < 0.05 and > 1 or < − 1 log_2_ foldchange (FC) were considered significantly upregulated (red) and down (blue) regulated respectively. MDP1 gene spot is indicated by a purple arrowhead. (**B**) Pathway analysis according to KEGG mapper classification showing top 10 enhanced (+ ve NES) or suppressed (-ve NES) pathways in MDP1-cKD BCG at day 4, 7 and 14. (**C**) Heat map showing common differentially expressed genes in MDP1-cKD BCG at day 4, 7 and 14 under functional categories. Color scale indicates differential regulation as transcript FC relative to the VC. Upregulation is indicated in orange, downregulation is blue. Heat map was generated using the pheatmap package^46^ on R software Version 2023.03.1 + 446^47^.

NAPs organize DNA into compact structures and influence transcription in response to environmental changes or stress^20–23^. As a NAP, we previously showed that MDP1 binds to DNA and causes genome compaction during stationary phase in *M. smegmatis*^26^. As such, insights on MDP1-cKD BCG transcriptome may provide clues on MDP1’s influence on gene regulation that might explain the observed phenotypes. To test this, we employed RNA sequencing to examine the global transcription profile of MDP-cKD BCG compared to VC with a focus on changes in gene expression as the bacilli traversed through lag, log, and early stationary phase (corresponding to day 4, 7 and 14 of in vitro culture). We considered differentially expressed genes (DEG) with adjusted p-value < 0.05 and log_2_fold-change of − 1 < or > 1 as significantly downregulated or upregulated respectively. MDP1 was significantly downregulated in MDP1-cKD BCG at all time points (Fig. 6A). However, MDP1_#2-cKD maintained an average of 3-fold MDP1 suppression compared to the VC at all time points, while MDP1 suppression in MDP1_#1-cKD altered with the lowest suppression (~2-fold) being at day 14. Regardless, 18 other genes were differentially expressed in both MDP1-cKD BCG and at all time points (Supplementary Fig. 6A).

Gene expression profile showed an increase in the number of DEG from lag to stationary phase, with more upregulated (81–240) than downregulated (5–175) genes in all growth phases for MDP1_#1-cKD and MDP1_#2-cKD compared to VC (Supplementary Fig. 6B). Based on the DEG locus, MDP1 seemed to exert a global reach in its regulation (Supplementary Fig. 6C,D). We validated the RNA seq results by RT-PCR (Supplementary Fig. 7). For comparison, we similarly observed more significantly upregulated genes at both log and stationary phase in *M. smegmatis* ∆MDP1 compared to wild type *M. smegmatis*. (Supplementary Fig. 8A,B). This shows that suppression of MDP1 results in upregulation of gene expression, which suggests that generally MDP1 is a gene repressor and it exerts its role in the cell by suppressing gene expression.

Pathway analysis showed that both MDP1-cKD BCG suppressed key biosynthetic pathways for growth and replication including the ribosome pathway and aminoacyl-tRNA biosynthesis corresponding to all time-points with low growth rates (Fig. 6B). Biosynthesis suppression might have led to transient downregulation (day 14) of molecular chaperones (*GroEL*, *GroES*, *DnaK, DnaJ)* (Supplementary Fig. 9A,B)*,* which are essential for maintaining protein structure and function in normal growth. These proteins are also essential in preventing damage and repairing proteins during stress^45^. Interestingly, *M. smegmatis* ∆MDP1 also suppressed the ribosome pathway at stationary phase, where it losses viability^25^, but not at log phase (Supplementary Fig. 8C,D). This observation suggests that the downregulation of macromolecular synthesis might be a contributing factor towards the low viability observed in MDP1-cKD BCG.

To further understand how MDP1 executes its roles through regulation of gene expression, we categorized DEG based on their functions (Fig. 6C). Transient differential regulation of transcriptional regulatory proteins indicates that MDP1 gene regulation might occur indirectly via its regulation of these transcription regulators. This might also explain how MDP1 manages to influence many widely dispersed genes within the genome (Supplementary Fig. 6C). Transcription regulators like sigma factors *sigI*, and *sigE* are involved in stress response. They control expression of many stationary phase associated genes that help *Mtb* survive prolonged stationary phase stress^48^ and their differential expression before stationary phase might be an indication of stress in MDP1-cKD BCG. This was further supported by the observed upregulation of the polyacyltrehalose (PAT) biosynthetic cluster genes (*pks3* (polyketide synthase), *papA3* (acyltransferase), *mmpL10* (lipid transport) and *fadD21)* (Supplementary Fig. 6A), which are typically upregulated during environmental stress including acidic and hypoxic stress^49^.

In addition to MDP1*,* we observed the differential expression of other genes involved in the maintenance of DNA integrity (*mutT1* and *recD*) (Fig. 6C). Also down regulated were genes involved in the maintenance of REDOX balance including *WhiB3*, rubredoxin A (*RubA*) and *RubB*. Also notable was the differential expression of *PE/PE_PGRS* genes which play a role in host-pathogen interactions by provoking pro-inflammatory or anti-inflammatory responses, and thus exhibit potential to act as molecular switches that can skew the responses as pro-host or pro-pathogen during Mtb infection^50^. Taken together, the differential expression of these genes indicates possible direct or indirect control by MDP1, and its suppression might have contributed to the susceptibility of MDP1-cKD to oxidative stress, lower infectivity and survival.

## Discussion

Gene fitness varies in different environments^43^. This has been illustrated in Mtb where out of approximately 614 in vitro essential genes^44^, only 194 genes are required for in vivo growth^43^. For this reason, we investigated the role of MDP1 in BCG survival in harsh conditions in vitro, and in vivo using a mice model. We illustrated suppression of MDP1 results in increased sensitivity of BCG to antibiotics (Fig. 2), oxidative stress (Fig. 3) and harsh host environments (Fig. 4). Low survival of MDP1-cKD BCG in mice resulted in deficient cytokine (IFN-γ and TNF-α) production by splenocytes (Fig. 5). Interestingly, in vitro temporal transcriptome analysis portrayed MDP1 as a gene repressor (Fig. 6), with possible control of genes involved in maintenance of DNA integrity, and adaptation to oxidative and host-related stress.

MDP1 is important for mycobacterial growth and is considered essential in pathogenic mycobacteria including BCG^20, 44^. Our attempts to acquire MDP1 knock out BCG, like others^18, 28^, were unsuccessful hence our choice to employ CRISPRi gene silencing system to study MDP1 functions in BCG. Targeting different MDP1 sites resulted in slightly different levels of significant MDP1 suppression in the two MDP1-cKD BCG, with MDP1_#2 exhibiting a stronger and more stable suppression (Fig. 6A). This variation is expected as the level of gene silencing by CRISPRi system varies depending on the position of the sgRNA target on the gene^40^. We speculate that this variance might also account for the differences in growth (Fig. 1B,C), susceptibility to drugs (Fig. 2B,C) and oxidative stress (Fig. 3B,C) as well as THP1 infectivity (Fig. 4A). Similar variation was also observed in a previous study where two Mtb constructs with >80% suppression of the essential gene *dfrA*, as quantified by qPCR, exhibited different sensitivities (Minimum Inhibitory Concentration of 75 µg/ml and 19.8 µg/ml) to methotrexate^40^. Nonetheless, both MDP1_cKD BCG exhibited significantly low growth tendencies correlating to MDP1 suppression compared to VC (Fig. 1B–D) which confirms MDP1 targeting. On the contrary, previous studies employing antisense gene silencing system to suppress MDP1 expression reported accelerated growth of recombinant BCG in vitro using ATP as a measure of growth^28^. This discrepancy might be associated with differences in gene silencing (antisense system achieves 50% suppression^28^ compared to 80% by CRISPRi^51^), and growth quantification method employed. We employed the gold standard CFU assay to quantify growth which is more robust than the ATP assay. Furthermore, we recorded no significant difference in CFU normalized ATP levels between MDP1-cKD BCG and VC (Supplementary Fig. 4). Regardless, unlike the previous study, our results prove the essentiality of MDP1 in BCG by showing that its suppression lowers BCG growth.

Like *M. smegmatis* ∆MDP1 (Supplementary Fig. 8), we observed an increase in the number of DEG (more up- than down regulated genes) in MDP1-cKD BCG as the bacteria grew from lag to stationary phase (Fig. 6A). We speculate that this change helps BCG to transition into the stressful stationary phase^25, 52, 53^. This is evidenced by the differential expression of stress related genes including transcription factors such as sigma factors, DNA repair and REDOX response genes^1, 41, 42, 48^ (Fig. 6C). It has been shown that in response to environmental cues, post-translational modification (acetylation and phosphorylation) of NAPs including MDP1 affects DNA binding, thus influencing nucleoid architecture and ultimately gene expression^21, 24, 30, 54, 55^. Taken together, our results indicate that as a NAP, MDP1 in BCG is a general gene repressor, and suggests that MDP1 is able to alter bacterial gene expression to aid in BCG adaptation to stress. As such, further studies to resolve the global binding capacity of MDPI using ChIP-based assays^21, 22^ will be useful in understanding the mechanism employed by MDP1 to tailor appropriate responses based on the environmental stress encountered.

While suppression of MDP1 did not result in alteration of ATP as was observed in *M. smegmatis* ∆MDP1^25^, MDP1 cKD BCG was susceptible to energy perturbations caused by bedaquiline (Fig. 2), which blocks ATP synthesis by binding to ATP synthase^1, 38^. In Mtb, delayed bactericidal effect of bedaquiline was shown to result from the ability of the bacilli to remodel its metabolism^1^. This might explain the bacteriostatic effect of bedaquiline on VC. Considering that MDP1 is upregulated in Mtb as a response to bedaquiline exposure^1^ and given the observed influence of MDP1 on gene expression, this metabolic remodeling ability might be lost with MDP1 suppression resulting in the increased killing of MDP1 cKD BCG. Furthermore, macromolecular synthesis pathways like ribosome are high energy consuming processes, and their suppression in MDP1-cKD (Fig. 6B) might be the bacterial response to maintain basal ATP levels required for basic cell maintenance, and could indicate inefficiencies in ATP production. A similar phenomenon was observed in *E. coli* which induces ribosome hibernation and reduced ribosome biogenesis as a strategy to modulate protein synthesis, and hence save energy during starvation and stress^56^. Taken together, this might explain the increased vulnerability of MDP1-cKD to energy perturbations caused by exposure to bedaquiline.

The maintenance of nucleoid structure and protection of mycobacterial DNA is one of the key roles played by MDP1^26, 57^. This role is particularly useful in the presence of oxidative stress as MDP1 prevents oxidative damage via Fenton reaction^41^, and its suppression might be one of the reasons for the low survival of MDP1-cKD under oxidative stress (Fig. 3). The suppression of *mutT1* might also contribute to this phenotype. *mutT1* codes for a nudix hydrolase protein that prevents genomic mutations and maintains the fidelity of protein synthesis under oxidative stress by hydrolyzing 8-oxo-G nucleoside triphosphates/diphosphates that were damaged by reactive oxygen species, to corresponding nucleoside monophosphates. As such, its suppression might contribute to accumulation of lethal genomic mutations. In fact, Mtb ∆*mutT1* mutants were shown to be highly sensitive to oxidative stress^58^.

We illustrated that MDP1 depletion instigated the suppression of REDOX related genes including *RubA, RubB* and *whiB3* (Fig. 6C). *RubA* and *RubB,* code for the iron-sulphur protein ruberodoxin which rapidly transfer metabolic reducing equivalents to oxygen or reactive oxygen species. They are preferably induced during oxidative stress since they are less affected by oxidative stress and only need one iron ion, which is especially useful in macrophage environment where iron is scarce^59^. On the other hand, *whiB3* proteins have been associated with a myriad of functions including sensing and responding to oxidative and nitrosative conditions. It was previously reported that *whiB3* expression is upregulated by exposure to H_2_O_2_ with Mtb ∆*whiB3* showing increased susceptibility to oxidative stress and low survival in macrophages^42^. This suggests that the suppression of these genes in MDP1-cKD might contribute to the low survival of BCG exposed to in vitro oxidative stress as well as in macrophages. It also implies that MDP1 directly or indirectly plays a role in BCG survival under oxidative stress, and corroborates similar study findings in Mtb^39^.

In a host-environment, we illustrated that compared to VC, fewer MDP1_#2-cKD BCG were able to infect both THP-1 macrophages (Fig. 5A) and establish infection in mice (Fig. 5B). Other studies had similar observations where fewer Mtb ∆MDP1 infected THP-1 and murine macrophages^27, 39^. This might be attributed to the lack of MDP1 mediation of the bacillary entry into host cells given its function as a surface adhesin^57^_._ In macrophages and in vivo environments, BCG is also likely challenged with nitrosative stress as well as nutrient deprivation. MDP1 is particularly critical for the regulation of iron homeostasis in the iron restricted intracellular environment^27, 60^ and its suppression might have also contributed to the low survival in THP1 and mice. Overall, our results are consistent with previous study results of MDP1 depletion resulting in low mycobacterial growth in blood monocytes, murine macrophage lines and PBMC^27–29^.

We acknowledge that employing only MDP1_2-cKD in the in vivo study is a limitation of our study. However, using MDP1_#2 cKD, which portrayed higher and more stable MDP1 suppression levels, we provide proof-of-concept that MDP1 plays a role in the survival of BCG in vivo. As the effectiveness of BCG depends on its viability^12, 13^, it is probable that low in vivo* survival* of MDP1_2-cKD inhibited prolonged immune stimulation resulting in deficient IFN-γ and TNF-α production by CD4^+^ T-cells from splenocytes (Fig. 5). This is informed by previous study findings that illustrated BCG clearance with antibiotics reduced the number of mycobacteria reactive effector cells in the spleen leading to gradual decrease in the organ immunity^61^. Aside from influencing viability, two previous studies—One using MDP1 suppressed BCG, and another employing a candidate booster vaccine consisting of MDP1 antigen in combination with CpG oligodeoxynucleotide (G9.1) as adjuvant—showed that MDP1 induces IFN-γ production in human PBMC^18, 62^, thus illustrating its immunogenicity. Furthermore, the probable influence of MDP1 on the expression of *PE/PE_PGRS* genes (Fig. 6C), which play a role in host-pathogen interactions, might have had an impact on the survival^42^ as well as the cytokine response induced by BCG. For instance, PE31 which was significantly upregulated in MDP-cKD has been shown to inhibit pro-inflammatory cytokines like IFN-γ^50^.

In summary, our detailed in vitro studies illustrate the significant role of MDP1 in BCG survival in harsh environments. For the first time, we showed that MDP1-dependent survival of BCG influences the immune response of CD4^+^ T cells from mice splenocytes. We also demonstrated that as a NAP, MDP1 is a general gene repressor. We envision that our work will initiate investigations on the specific roles of MDP1 in BCG immune regulation that can be useful for the development of new vaccine interventions. Furthermore, given MDP1’s versatility in the regulation of gene expression in BCG, our study offers additional evidence and reasoning to target MDP1 for drug development not only for BCG’s parental strain and Mtb, but other pathogenic mycobacteria (e.g. *M. leprae*) which share striking sequence similarities^20, 26^.

## Methods

### In vitro studies

#### Bacterial strains and general growth conditions

Liquid cultures were grown in Middlebrook 7H9 broth (BD, Franklin Lakes, NJ) supplemented with 0.2% (v/v) glycerol, 0.05% (v/v) Tween 80 (MP Biomedicals, Santa Ana, CA), and 10% ADC enrichment (5% bovine serum albumin [Wako Pure Chemical Industries, Osaka, Japan], 0.81% NaCl, and 2% D-glucose) (7H9-ADC broth). Solid cultures were grown on Middlebrook 7H10 agar (BD) supplemented with 0.5% (v/v) glycerol and 10% OADC enrichment (ADC enrichment supplemented with 0.06% [v/v] oleic acid) (7H11-OADC agar). To maintain strain genotype BCG cultures were supplemented with 20 μg/ml kanamycin (Km) and 50 μg/ml hygromycin (Hyg) (Wako Pure Chemical Industries (Osaka, Japan) for selective growth. All cultures were incubated at 37 °C.

#### Strain construction

CRISPR interference system was used to silence MDP1 expression as previously reported^26, 40^. pRH2502, a vector expressing an enzymatically inactive Cas9 (dCas9), and pRH2521, a vector expressing small guide RNAs (sgRNA), were gifted by Dr. Robert N. Husson^40^. The dCas9 and sgRNA expression was regulated by TetR-promoters (uvtetO and Pmyc1-tetO, respectively). To conditionally knockdown MDP1 in BCG Tokyo 172, two sgRNAs were designed to target the non-template strand of MDP1(#1; CCGCCGTCGAGAATGTCGTT, #2; GCAATCCGCGTACCGGCGAG). To minimize off-target effect, we confirmed that there was no gene which had similar sequence to sgRNA with less than 5 mismatches by a BLAST search. Each designed oligonucleotide for sgRNA expression was ligated into pRH2521 (pRH2521-MDP1#1 and pRH2521-MDP1#2). These vectors were introduced into BCG cells containing the pRH2502 (for dCas9 expression) integrating vector, and then strains selected on agar plates containing 50 μg/ml Hyg and 20 μg/ml Km. For induction of dCas9 and sgRNA expression (i.e. knockdown of MDP1), anhydrotetracycline (ATc) (Cayman chemical company, Ann Arbor, MI) was supplemented to in vitro bacterial culture at a final concentration of 200 ng/ml. In vivo, doxycycline (Doxy) (Kyoritsu pharmacy, Japan) was supplied in mice drinking water at a final concentration of 20 µg/ml.

#### Bacterial culture

Bacteria were cultured as previously described^26, 63^ with minor adjustments. BCG from mid-logarithmic phase were inoculated into 7H9/ADC supplemented with selective antibiotics to an initial OD_600_ of 0.025 and incubated in microaerobic standing cultures at 37 °C. Once the culture OD_600_ reached 0.1, ATc was added every other day during the chase period and aliquots harvested to determine OD_600_ values, viability, and protein expression.

In vitro viability was assessed using the colony forming unit (CFU) assay^64^. At indicated time points 100 µl of each culture was harvested, and 10-fold serial dilutions were made with Saline Tyloxapol Buffer (STB) to disrupt bacteria clots^65^ before plating 10 µl on Middlebrook 7H10/OADC agar supplemented with appropriate antibiotics. The plates were then incubated at 37 °C for 3 weeks after which colonies were counted from dilutions that yielded 20–100 colonies and CFU/ml values calculated.

Protein expression was confirmed using western blot analysis exactly as previously described^26^. For our study purpose MDP1 protein was detected with mouse monoclonal anti-MDP1 antibody (10,000^–1^) as primary antibody, and horse radish peroxidase (HRP)-conjugated rabbit anti-mouse immunoglobulins (5000^-1^) as secondary antibody. On the other hand, *InhA* protein was detected using anti-*InhA* (5000^–1^) as primary antibody, and donkey anti-rabbit immunoglobulins (5000^–1^) as secondary antibody.

#### Measurement of ATP level

Culture ATP levels at indicated time points were measured using the BacTiter-Glo^TM^ Microbial cell viability (Promega, Madison, WI, USA) assay kit following manufacturer’s instructions with minor adjustments. One hundred microliter of the culture was mixed with 15 µl of the BacTiter-Glo^TM^ reagent per assay in a 96-well black plate. Luminescent signal and hence the amount of ATP present was detected using luminometer (Filter Max F5 Multi-Mode Microplate reader) with the SoftMax Pro Easy software and quantified as relative light units (RLU).

#### Drug sensitivity

BCG sensitivity to bedaquiline and rifampicin was assessed as previously described^29, 38^. After confirmation of MDP1-cKD, culture OD_600_ was adjusted to 0.2 and further diluted 1:100 in fresh medium to a final estimated OD of 0.002. The cultures were then exposed to varying concentrations (0.01, 0.03, 0.06, 0.125, 0.25µg/ml) of bedaquiline or rifampicin in 96 well microtiter plates and incubated at 37 °C normoxia with ATc supplementation after every 48 hours. These were chased for 2 weeks. Aliquots were harvested at indicated time points for CFU quantification.

#### Analysis of sensitivity to oxidative stress

BCG liquid cultures were grown to logarithmic phase. Their OD_600_ was adjusted to 0.2 and further diluted 1:10 in fresh medium for a final estimated culture OD of 0.02. The cultures were exposed to H_2_O_2_ (100 µM, 1 mM and 5mM) and menadione (20, 50, 100 µM) in 96 well microtiter plates incubated at 37 °C normoxia^25^. ATc was added at day 0 and the cultures chased for 48 hours. Aliquots were harvested at indicated time points for CFU quantification.

#### Intracellular survival in THP1 macrophage cell line

Differentiated THP-1 cells were infected with VC and MDP1-cKD BCG (after confirming MDP1 expression) at an MOI of 1:1 for 4 hours^63^. Uninfected BCG were washed away twice using warm serum free Dulbecco’s modified Eagle’s medium (DMEM). Cultures were incubated at 37 °C in a humidified 5% CO_2_ incubator and supplemented with ATc. At indicated timepoints, macrophages were disrupted using 0.5% Triton X-100, and the lysate diluted and spread on 7H10/OADC agar for eventual CFU determination.

#### RNA extraction from in vitro cultures and quality control

RNA was extracted, using Direct-zol^TM^ RNA MiniPrep Plus kit (Zymo Research, Irvine, CA) from VC and MDP1-cKD BCG as well as *M. smegmatis* WT and *M. smegmatis* ∆MDP1 cultures at indicated time points following the manufacturer’s instructions. Extracted RNA was quantified by spectrophotometry using BioSpectrometer and initial quality assed using gel electrophoresis. DNA contamination was checked by running PCR amplification products on 1% agarose gel electrophoresis^26, 63^. RNA integrity (RIN) of samples with A_260/280_ and A_260/230_ above 1.8 was checked using the TapeStation (Agilent) and samples with RIN values above 7 and rRNA ratio above 0.8 proceeded for RNA sequencing by Macrogen Japan. RNA seq was performed using the Novaseq 6000 illumina sequencing platform at 100bp paired end reads. Validation of the RNA seq data was done using quantitative real-time PCR assay (qRT-PCR) by quantifying *hupB*, *mutT1*, *Pks3* and *narK1* expression using primers indicated in Supplementary Table 1. Simply, ReverTraAce qPCR-RT Master mix with gDNA remover (Toyobo, Japan) was used to synthesize cDNA following the manufacturer’s instructions. qRT-PCR reaction mixtures were then prepared using THUNDERBIRD Taqman qPCR Mix (Toyobo, Japan) according to the manufacturer’s instructions. The qRT-PCR reactions were performed using the CFX connect Real Time System (Bio-Rad laboratories, Hercules, CA). Relative gene expression was determined by a calculated threshold cycle (CT) and data normalized against *sigA* and 16S rRNA as internal standards.

### In vivo studies

#### Ethical statement

All animal experimental procedures and housing conditions were reviewed and approved by the Animal Care and Use Ethics Committee (Permit number: SA0050503 and SA00929) of Niigata University Graduate School of Medicine. All animals were cared for and treated humanely in accordance with the Institutional Guidelines for Experiments Using Animals. Animals were housed and maintained in specific-pathogen-free conditions at ABSL-2 facility at Niigata University Animal Centre. All experiments complied with the ARRIVE guidelines.

#### BCG survival in mice

After confirming MDP1 suppression *in vitro*, female C57BL/6JJc1 mice (5-8 per group) aged between 6 and 8 weeks (Japan Clea, Suita, Osaka, Japan), were intraperitoneally infected with 5 × 10^6^ CFU of VC and MDP1_#2-cKD BCG in 200µl saline^66^. The control group was injected with saline. The following day three mice were anesthetized using an anesthesia cocktail of Medetomidine (Orion Pharma, Finland), Midazolam (Sandoz, Novartis) and Butorphanol (Meiji, Japan) at 0.1 ml/10 g of mice, and sacrificed by cervical dislocation. To confirm infection load mice spleen, lung, and liver were harvested and homogenized using gentleMACS^TM^ dissociator (Multenyi Biotec). The homogenate was then serial diluted and plated on 7H10/OADC agar with relevant antibiotics^67, 68^. The plates were incubated for 3 weeks at 37 °C after which BCG colonies were enumerated and CFU per organ calculated^63^. The same protocol was repeated at 28 days post infection.

#### Immune response to BCG immunization

##### Preparation of splenocyte single cell suspensions

At 4-weeks post-intraperitoneal immunization with 5x10^6^ CFU of VC, MDP1_#2-cKD BCG or 200 µl saline, mice (4–8 mice per group) were sacrificed, and spleens gently ground and filtered through a 70 µm cell strainer. Red blood cells were lysed by 0.83% NH_4_Cl solution followed by two washes with PBS and filtration. This was followed by preparation of single-cell suspensions using Lympholyte-M (Cedarlane Laboratories, Bulington, NC) via density-gradient centrifugation procedure following the manufacturer’s instructions. Harvested single-cell suspension were resuspended in RPMI-1640 medium (FUJIFILM Wako pure Chemical Corporation, Japan) containing 10% FCS, 2 mM glutamine, 50 µM β-mercaptoethanol, 100 µg/ml streptomycin and 100 U/ml penicillin^66^.

##### In vitro peptide stimulation

Single cell suspensions were adjusted to 2×10^6^ cells/ml and re-stimulated with purified protein derivative (PPD, 5 µg/ml)^67^ or phorbol 12-myristate 13-acetate (PMA)/ionomycin (25 ng/ml and 1µg/ml, as flow cytometry positive control) all in the presence of co-stimulatory antibody (anti-CD28/CD49d - 0.25 µg/ml). No stimulation negative control was also included.

#### ELISA IFN-γ analysis

In flat-bottomed 96 well plates 2 × 10^5^ cells/well were incubated with respective stimulants at 37 °C, 5% CO_2_, 100% humidity for 3, 5 and 7 days. Culture supernatants were then harvested and subsequently used to quantify IFN-γ using the mouse IFN-γ ELISA kit (Biolegend, San Diego, CA), following the manufacturer’s instructions.

#### Surface and intracellular cytokine staining for flow cytometry

Approximately 2 × 10^6^ cells/well were incubated with respective stimulants and anti-CD49d in 24 well plates at 37 °C, 5% CO_2,_ 100% humidity for 12 hours. Brefeldin A (10 µg/ml) (BD Biosciences, Japan) was added after the first 7 hours followed by a further 5 hours incubation^66^. Cells were then washed with staining buffer (SB - 2% FCS, 0.05% NaN3 in PBS) twice. To reduce background, cells were treated with FcR block Ab (2µl/1ml in SB) (BD Biosciences) for 5 min at 4 ℃. They were then washed twice with PBS then stained with Fixable viability dye (FVD) eFlour 506 (1 µl/ml) (Invitrogen) for 30min at 4 ℃. Next was two washing steps with SB, then surface staining in 100 µl SB using anti-mouse CD3 (2 µl/100µl), anti-mouse CD4 (1 µl/100µl), anti-mouse CD8 (1 µl/100µl) (Tonbo biosciences) incubated for 30 min at 4 ℃. Surface-stained cells were then fixed and permeabilized using the Fixation/ Permeabilization solution (BD Biosciences) following the manufacturer’s instructions followed by intracellular cytokine staining with anti-mouse IFN-γ (1 µl/100µl) (Invitrogen eBiosciences), anti-mouse IL2 (1 µl/100µl) (Invitrogen eBiosciences) and anti-mouse TNF-α (1 µl/100µl) (Biolegend)^67^. Cells were analyzed using the Acea NovoCyte 3000 flow cytometer and gated using the NovoExpress 1.3.0 software.

### Statistical analysis

Results are presented as mean ± standard error (SE). Unpaired Welch’s t-test was used to test for significance between groups for OD, CFU and ATP assays. Mann-Whitney U test was used to analyze the difference between BCG immunized mice groups. Kruskal wallis test and dunn post-hoc test was used to analyze the significance in MFI between groups from flow cytometry analysis and ELISA. R software^47^ was used for all analysis and P < 0.05 was considered significant. For RNA sequencing analysis, DESeq2 was used to normalize the number of reads for each gene by relative log expression (RLE) conversion, and significant differences for each gene was calculated. Pathway analysis was done using the clusterProfiler package^69^ and KEGG database on R software.

### Supplementary Information

Supplementary Information.

## Data Availability

The original contributions presented in the study are included in the article/Supplementary material. RNA seq data was deposited in NCBI’s Gene Expression Omnibus and are accessible through GEO Series accession number GSE222247 (https://www.ncbi.nlm.nih.gov/geo/query/acc.cgi?acc=GSE222247). Further inquiries can be directed to the corresponding authors.
