# Supplementary material for: Mycobacterial DNA-binding protein 1 is critical for BCG survival in stressful environments and simultaneously regulates gene expression
Source: Sci Rep. 2023 Aug 29;13:14157. doi: 10.1038/s41598-023-40941-9 (PMC10465568; doi:10.1038/s41598-023-40941-9)
Supplement: Supplementary file 1 — Supplementary Information. [file 41598_2023_40941_MOESM1_ESM.pdf]

Supplementary Information

Mycobacterial DNA-binding protein 1 is critical for BCG survival in stressful environments and simultaneously regulates gene expression

Amina K. Shaban*, Gebremichal Gebretsadik, Mariko Hakamata, Hayato Takihara, Erina Inouchi, Akihito Nishiyama, Yuriko Ozeki, Yoshitaka Tateishi, Yukiko Nishiuchi, Takehiro Yamaguchi, Naoya Ohara, Shujiro Okuda, and Sohkichi Matsumoto* Correspondence and requests for materials should be addressed to A.K.S (email: kaboso90@gmail.com) and S.M. (email: sohkichi@med.niigata-u.ac.jp).

Supplementary data summary:

Supplementary Figure 1. Growth and MDP1 expression in cultures supplemented with ATc or DMSO. Page 3

Supplementary Figure 2. Full length western blot images showing MDP1 suppression in cultures supplemented with ATc and not DMSO. Page 5

Supplementary Figure 3. Full length western blot images confirming MDP1 expression in cultures supplemented with ATc. Page 7

Supplementary Figure 4. Similar ATP levels between VC and MDP1-cKD BCG. Page 8

Supplementary Figure 5. BCG promotes cytokine production in $CD4^+$ than $CD8^+$ T cell. Page 9

Supplementary Figure 6. Differentially expressed genes in MDP1-cKD BCG and their location. Page 10

Supplementary Figure 7. RNA sequencing validation by real-time PCR. Page 11

Supplementary Figure 8. Global transcription response to MDP1 depletion in *M. smegmatis*. Page 12

Supplementary Figure 9. MDP1 suppression in BCG influences expression of molecular chaperone genes. Page 14

Supplementary Table 1. qRT-PCR primer sequences used in this study. Page 16

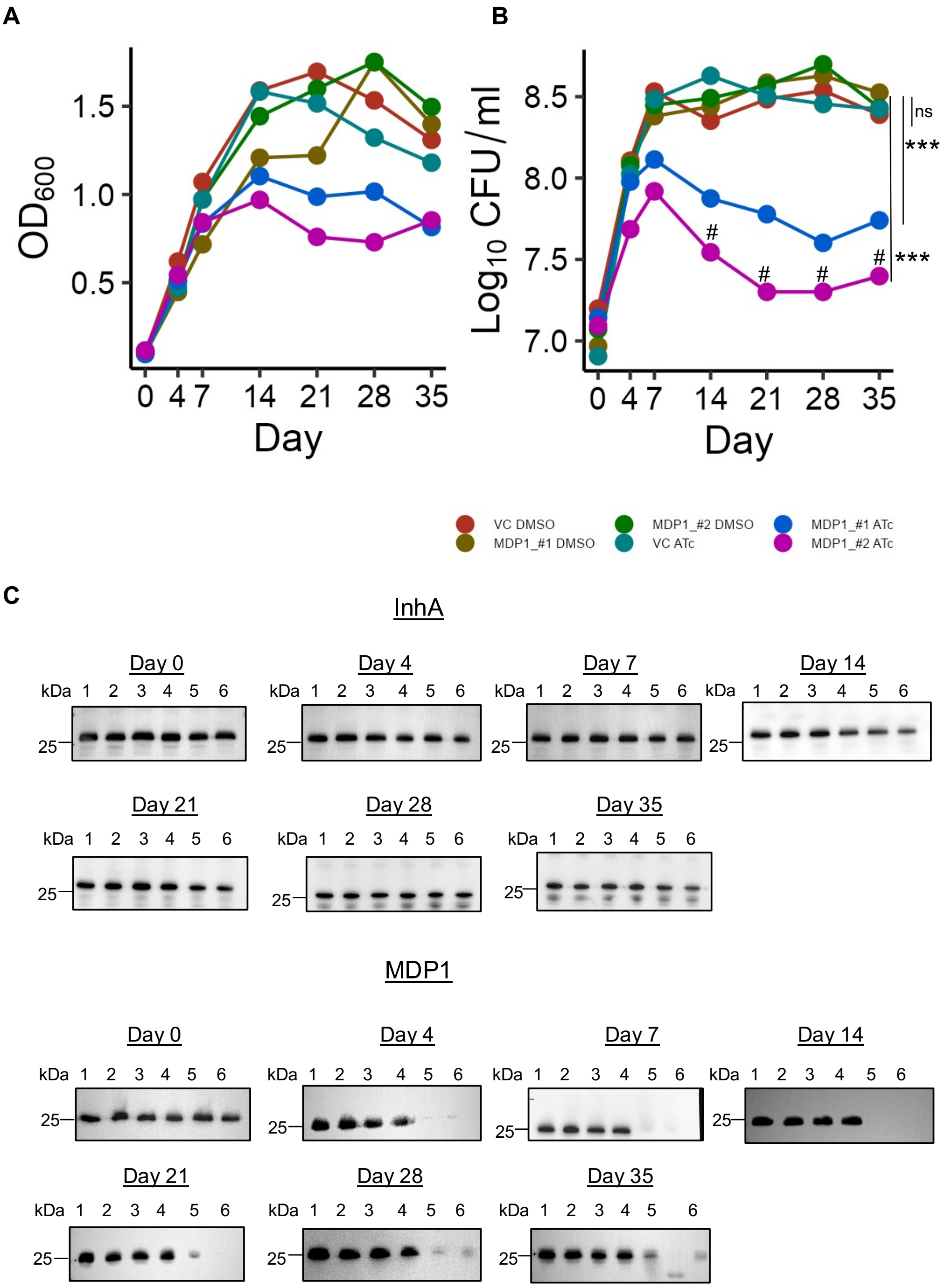

Supplementary Figure 1. Growth and MDP1 expression in cultures supplemented with ATc or DMSO. Growth kinetics of indicated BCG strains over 35 days depicted by **(A)** opacity density (OD_{600} nm) and **(B)** CFU for viability quantification. Bacteria were cultured in 7H9/ADC medium supplemented with 200ng/ml ATc or an equal volume of DMSO every 48 hrs. At indicated time points culture aliquots were harvested and used to determine the OD and

enumerate CFU after culturing on 7H10 agar. Data represent mean from two technical replicates. Statistical differences between VC ATc and MDP1-cKD BCG (indicated by *) or between MDP1-cKD clones (indicated by # where $p<0.05$) were assessed by unpaired Welch's T-test, *P<0.05 **P<0.01 ***P<0.001 ns=not significant. **(C)** Representative western blot image cropped from different gels delineated with black border lines confirming MDP1 expression for 1-VC DMSO, 2-MDP1_#1 DMSO, 3-MDP1_#2 DMSO, 4-VC ATc, 5-MDP1_#1 ATc and 6-MDP1_#2 ATc. *InhA* was used as a loading control. Full western blot images are shown in **Supplementary Fig. 2**.

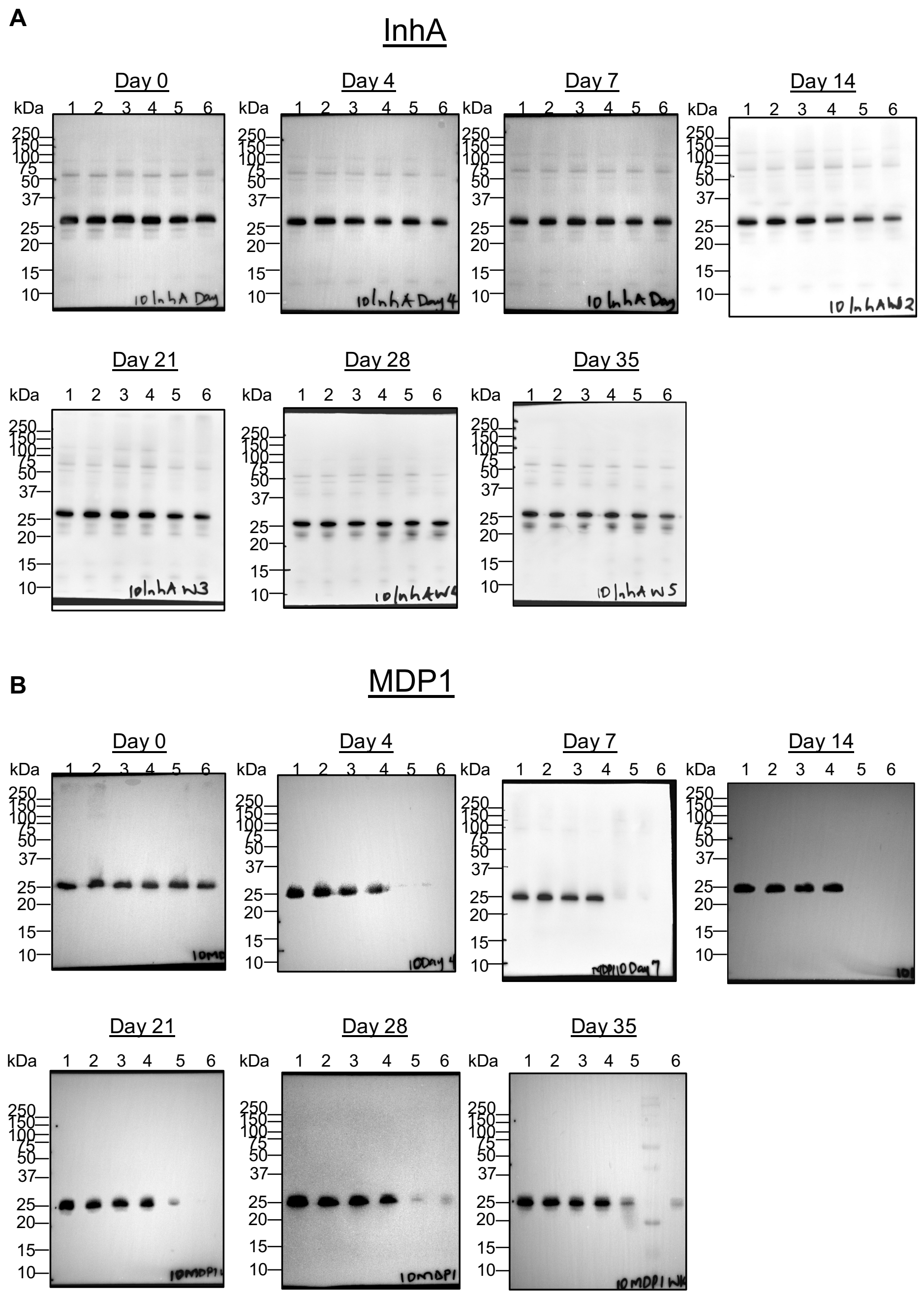

Supplementary Figure 2. Full length western blot images showing MDP1 suppression in cultures supplemented with ATc and not DMSO. Bacteria were cultured in 7H9/ADC medium supplemented with 200ng/ml ATc every 48 hours to induce sgRNA expression. At indicated time points culture filtrates were used to detect **(A)** *InhA* and **(B)** MDP1 proteins. *InhA* was used as a loading control. Positions of molecular weight markers are shown on the left side of each panel. 1-VC DMSO, 2-MDP1_#1 DMSO, 3-MDP1_#2 DMSO, 4-VC ATc, 5-MDP1_#1 ATc and 6-MDP1_#2 ATc. Image is representative of two replicates.

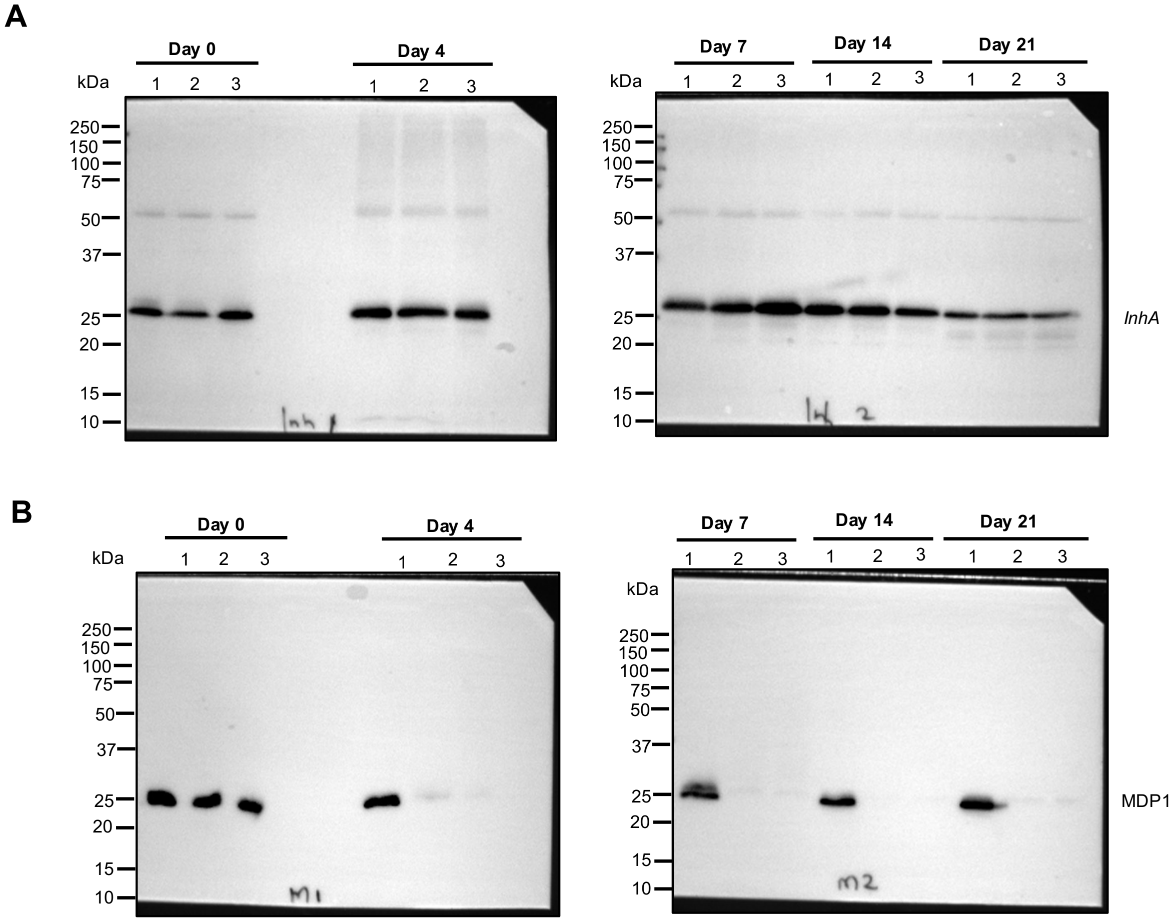

Supplementary Figure S3. Full length western blot images confirming MDP1 expression in cultures supplemented with ATc. Bacteria were cultured in 7H9/ADC medium supplemented with 200ng/ml ATc every 48 hours to induce sgRNA expression. At indicated time points culture filtrates were used to detect **(A)** *InhA* and **(B)** MDP1 proteins. *InhA* was used as a loading control. Positions of molecular weight markers are shown on the left side of each panel. 1-VC aTC, 2-MDP1_#1-cKD, 3-MDP1_#2-cKD. Image is representative of three experiments.

A

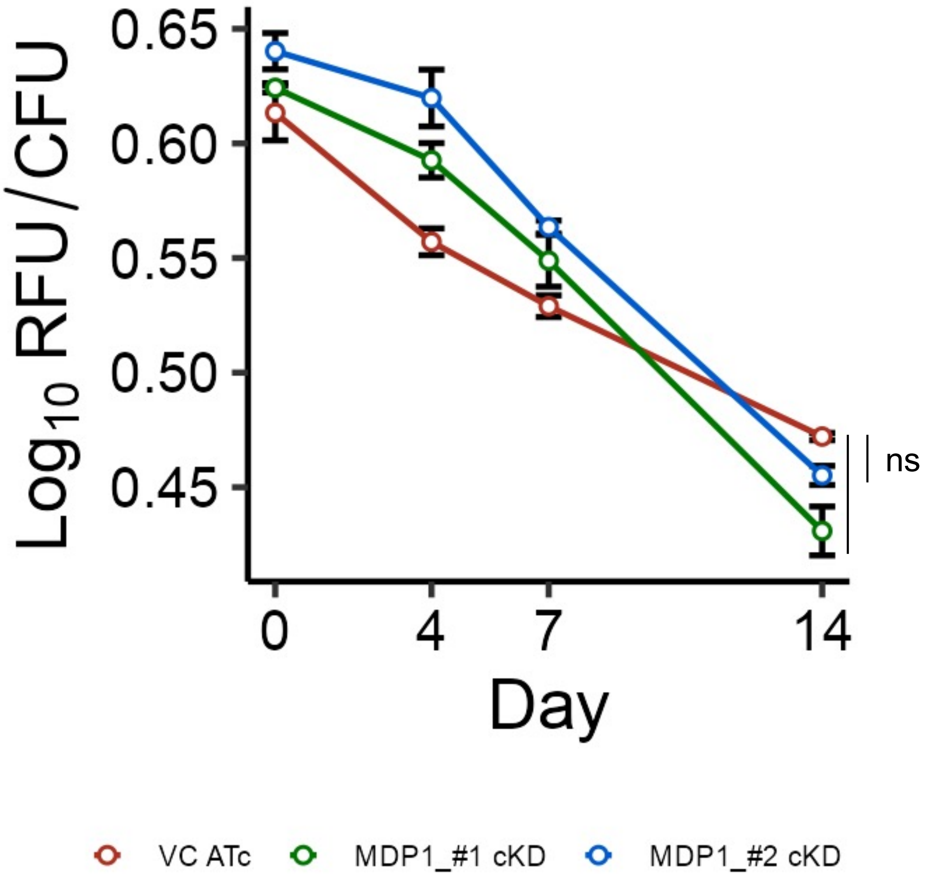

Supplementary Figure 4. Similar ATP levels between VC and MDP1-cKD BCG. **(A)** Effect of MDP1 on ATP production. Bacteria were cultured in 7H9/ADC medium supplemented with 200ng/ml ATc every 48 hours to induce sgRNA expression. At indicated time points intracellular ATP was quantified using the BacTiter-Glo microbial cell viability assay kit (Promega). The detected reflective light units (RLU) were normalized by CFU. Data represent mean ± SE from three biological replicates. Statistical differences between VC ATc and MDP1 cKD BCG were assessed by unpaired Welch's T-test, ns= not significant.

A

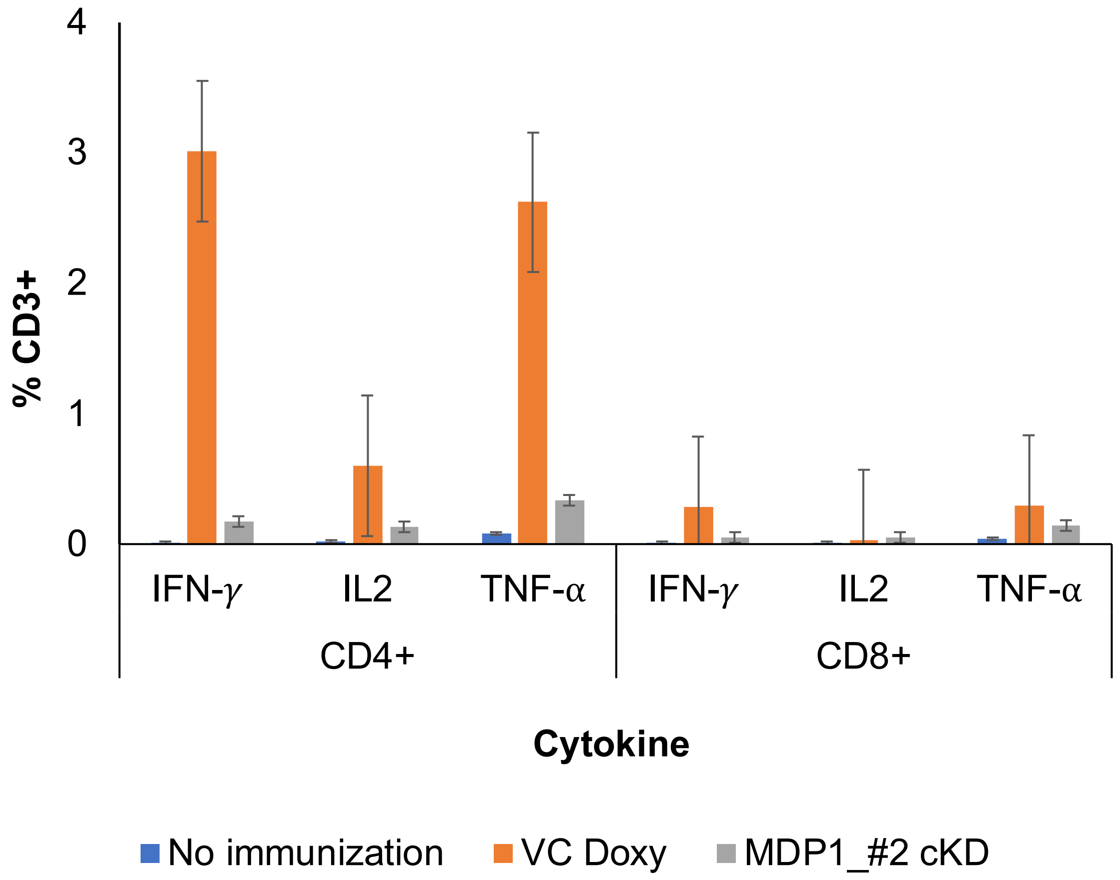

Supplementary Figure 5. BCG promotes cytokine production in $CD4^+$ than $CD8^+$ T cell. (**A**) Percentage of $CD3^+$ T cells population ($CD4^+$ and $CD8^+$) producing cytokines (IFN-γ, IL2 and TNF-α) in mice 4 weeks post immunization with $5x10^6$ CFU of BCG VC, MDP1_#2 cKD or saline for the no immunization control group. Results shown as mean $\pm$ SE from 4-6 mice per group.

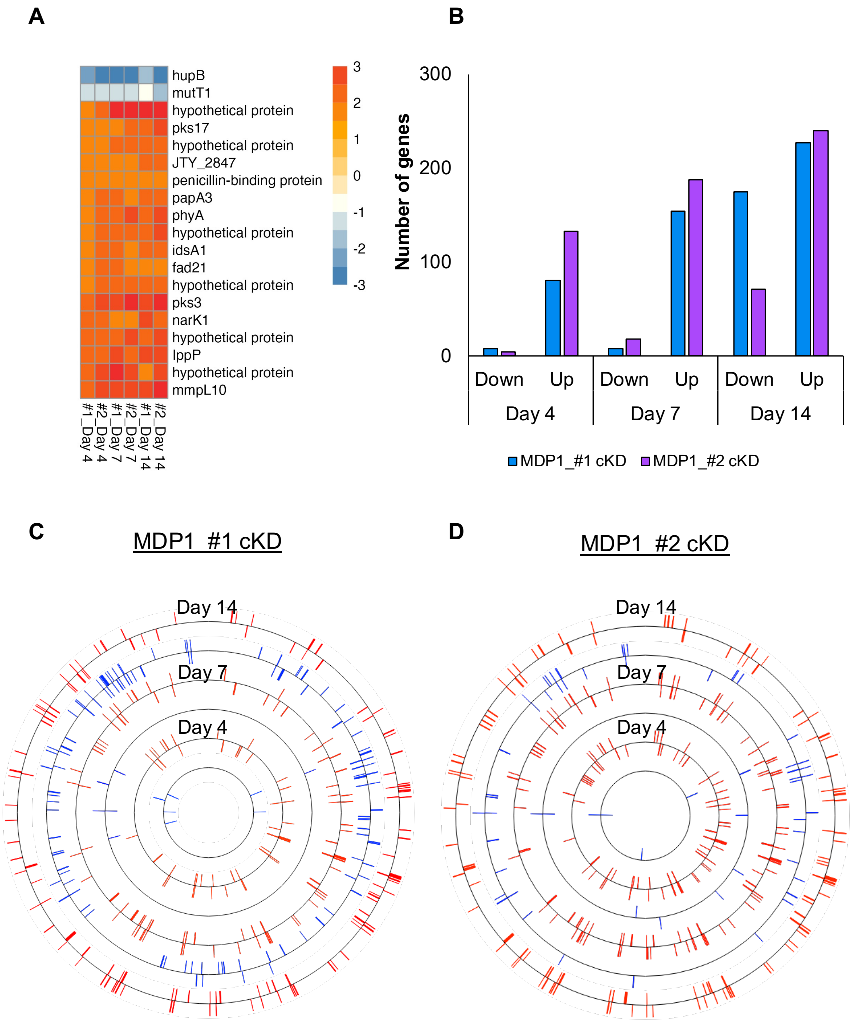

Supplementary Figure 6. Differentially expressed genes in MDP1-cKD BCG and their location. **A)** Heat map showing common differentially expressed genes in both MDP1 cKD BCG and in all the indicated time points. Color scale indicates differential regulation as transcript FC relative to the VC. Upregulation is indicated in orange, downregulation is blue. (**B**) The number of significantly upregulated and downregulated genes in MDP1_#1 cKD and MDP1_#2 cKD BCG compared to VC ATc at day 4, 7 and 14. Genes with adjusted p-value<0.05 and >1 or <-1 log_2foldchange (FC) were considered significantly upregulated and down regulated respectively. Genome map showing the gene loci of differentially expressed genes in **C)** MDP1_#1 cKD **D)** MDP1_#2 cKD at day 4, 7 and 14. Red and blue lines indicate upregulated and downregulated regulated genes, respectively. Genome map generated using BioCyc genome database collection software.

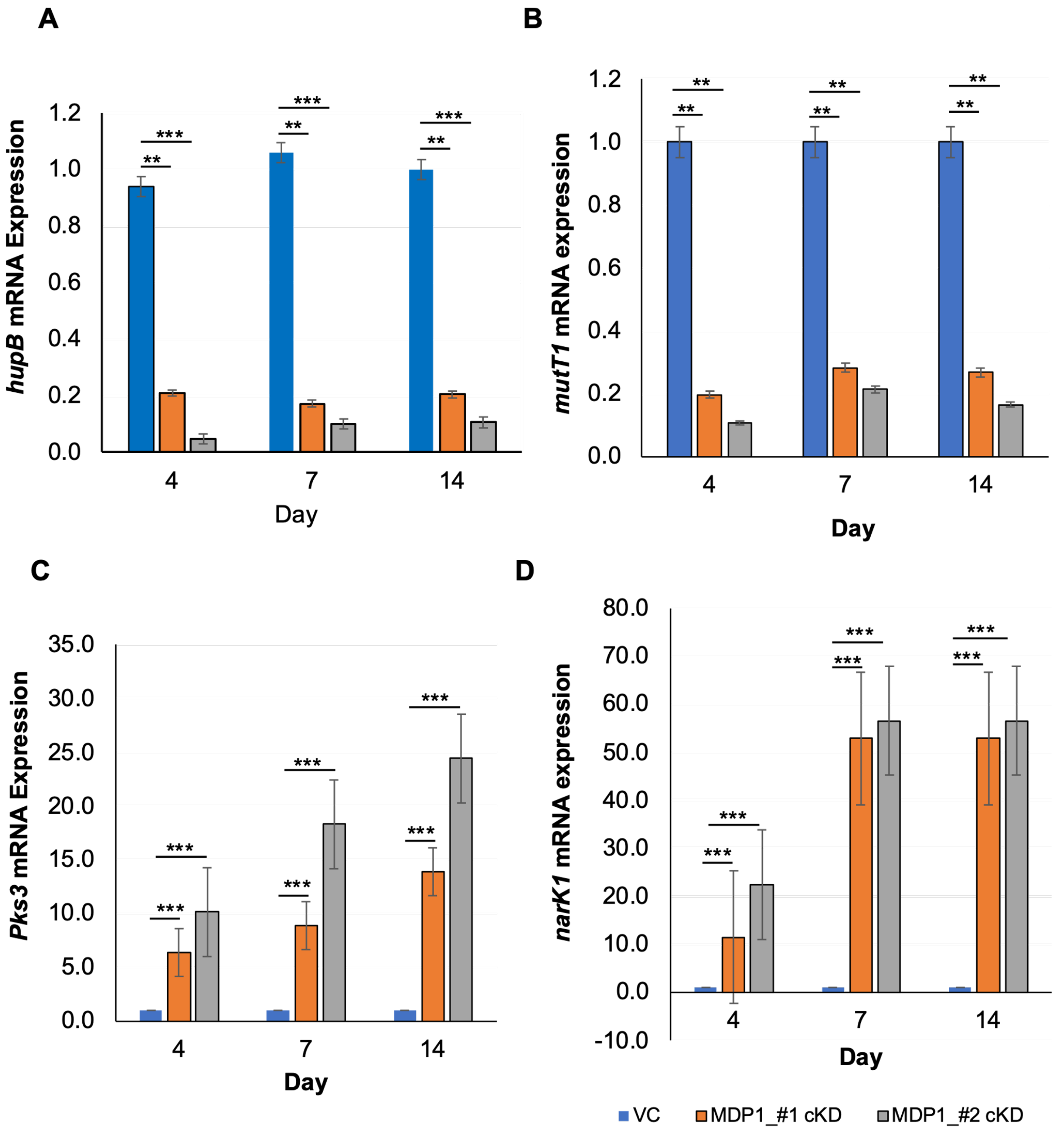

Supplementary Figure 7. RNA sequencing validation by real-time PCR. mRNA quantification of **(A)** *hupB*, **(B)** *mutT1* **(C)** *pks3* **(D)** *narK1* in VC and MDP1 cKD BCG at day 4, 7 and 14. Data presented as mean + SD from triplicate experiments. Statistical significance was determined using one-way ANOVA followed by Dunnett's multiple comparison test, **P<0.01 ***P<0.001

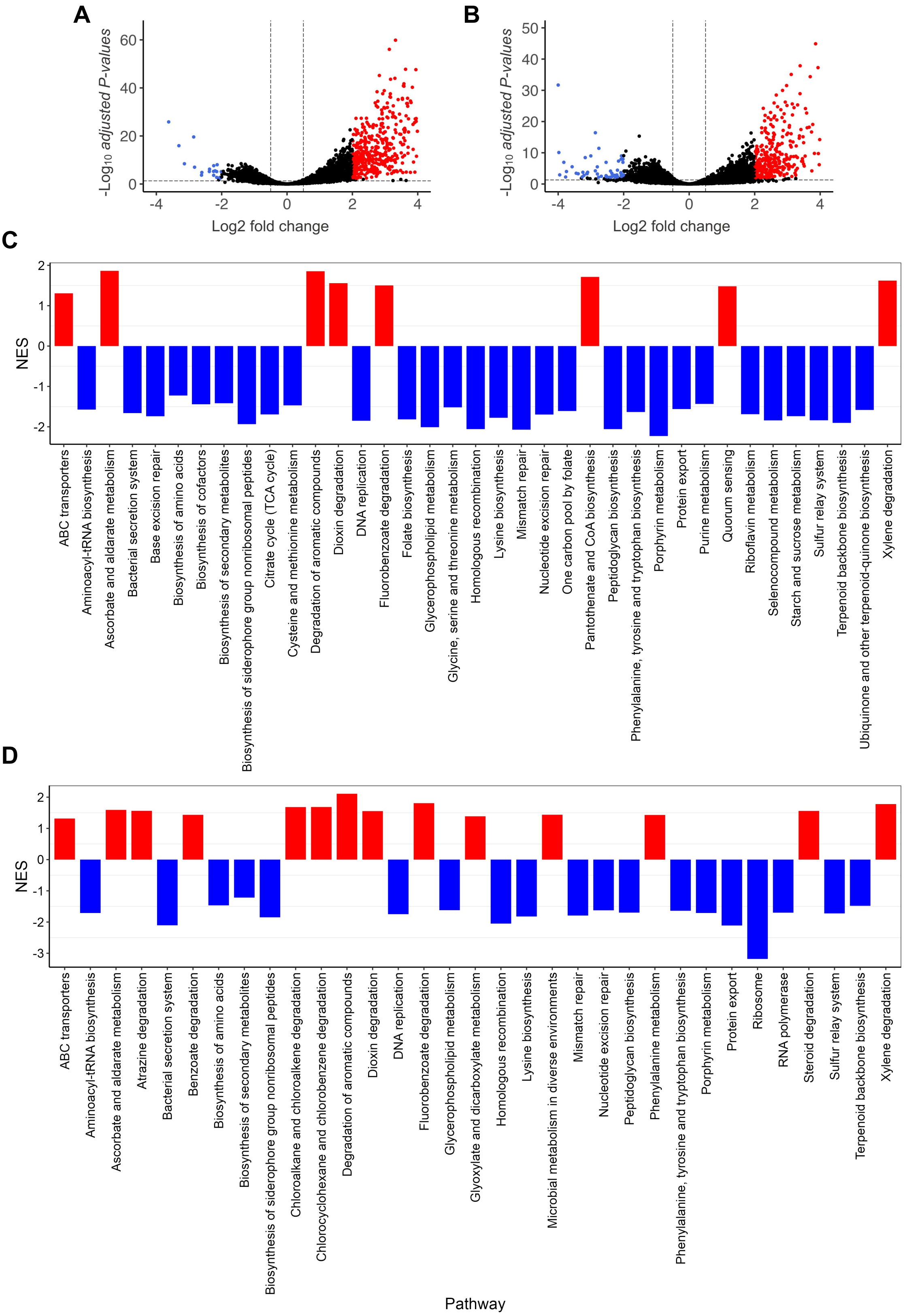

Supplementary Figure 8. Global transcription response to MDP1 depletion in *M. smegmatis*. Genome wide gene expression changes at **(A)** log and **(B)** stationary phase in *M. smegmatis* ΔMDP1 compared to wild type *M. smegmatis* from biological triplicate experiments. Genes

with $p<0.05$ and >2 or <-2 log_2foldchange (FC) were considered significantly upregulated (red) and down (blue) regulated respectively. Pathway analysis based on normalized enrichment score (NES) according to KEGG mapper classification showing enhanced (blue bars) or suppressed (red bars) pathways in *M. smegmatis* ΔMDP1 at **(C)** log and **(D)** stationary phase.

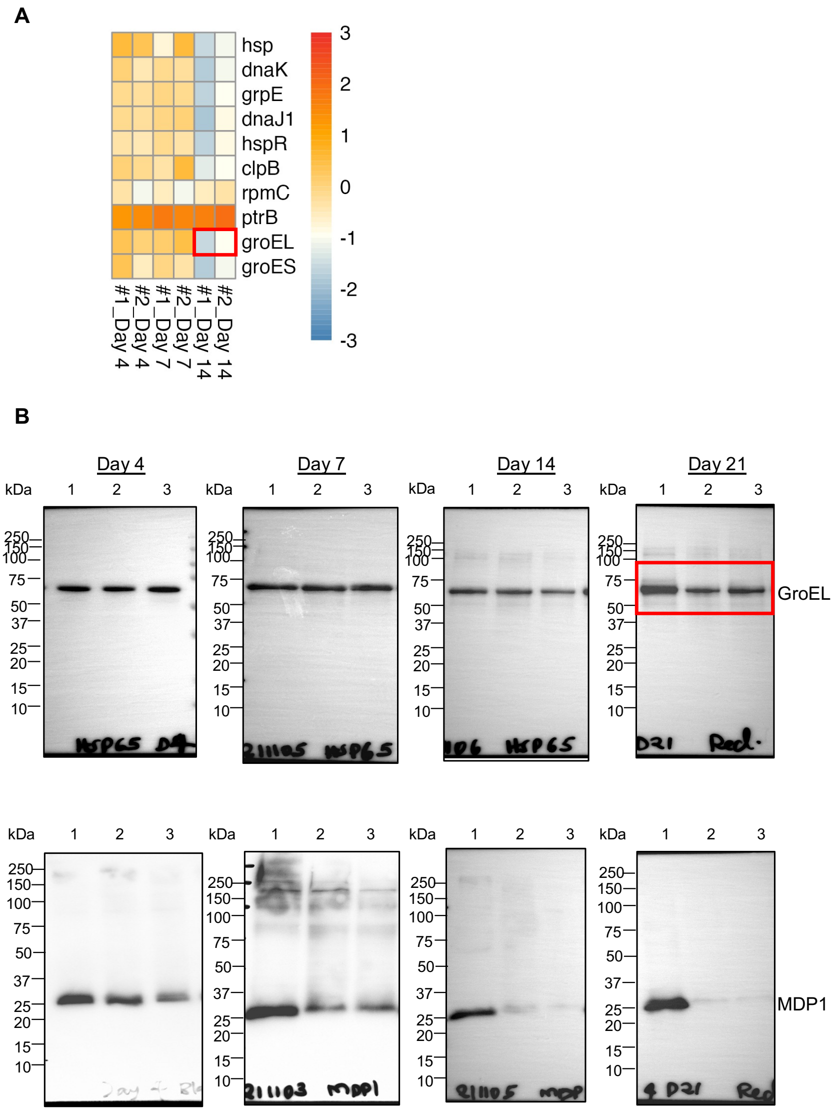

Supplementary Figure 9. MDP1 suppression in BCG influences expression of molecular chaperone genes **(A)** Heat map showing expression of molecular chaperone genes in both MDP1-cKD BCG at day 4, 7 and 14 from biological triplicate experiments. The color scale indicates differential regulation as mean transcript FC relative to the VC. Upregulation is indicated in orange, downregulation is blue. Transiently downregulated genes are indicated in red. (**B**) Full western blot images confirming the expression of *GroEL* and MDP1 at indicated timepoints for 1-VC ATc, 2-MDP1_#1 cKD and 3-MDP1_#2 cKD. Transient suppression of *GroEL* protein levels in MDP1 cKD BCG is highlighted in red. Positions of molecular weight markers are shown on the left side of each panel. Image is representative of biological duplicate experiments.

Supplementary Table 1. qRT-PCR primer sequences used in this study.

Target	Position	Sequence
sigA	Forward	5'-CCCACCGAAAAGGACAAGGC-3'
	Reverse	5'-GCCGATCTGTTTGAGGTAGGC-3'
16s	Forward	5'-TCGTGTCGTGAGATGTTGGG-3'
	Reverse	5'-CATCGCAGCCCTTTGTACCG-3'
hupB	Forward	5'-TTACTGTCTCGCCGGTACGC-3'
	Reverse	5'-CGACAGCGTCACCATTACCG-3'
mutT1	Forward	5'-ACGAGCTGATCTGGTTACCG-3'
	Reverse	5'-ACGACCCCTCTTGTCTAGCG-3'
pks3	Forward	5'-ATCGCGGTGTATCAGCAAGCG-3'
	Reverse	5'-ATTCGACCGGATCTCCAACGG-3'
narK1	Forward	5'-CGCAGCGCCATATTGATTCCG-3'
	Reverse	5'-CGCGTTGTTCGTCCTATCCG-3'
